# Subgroup analyses and patterns of multiple sclerosis health service utilisation: A cluster analysis

**DOI:** 10.1177/20552173241260151

**Published:** 2024-06-20

**Authors:** Lara Marleen Fricke, Kathrin Krüger, Corinna Trebst, Anna Levke Brütt, Elise-Marie Dilger, Kerstin Eichstädt, Peter Flachenecker, Anja Grau, Melissa Hemmerling, Dyon Hoekstra, Kristina Schaubert, Alexander Stahmann, Jona Theodor Stahmeyer, Annett Thiele, Uwe Klaus Zettl, Fedor Heidenreich, Christian Krauth

**Affiliations:** Hannover Medical School, Institute for Epidemiology, Social Medicine and Health Systems Research, Hannover, Germany; Center for Health Economics Research Hannover (CHERH), Hannover, Germany; Hannover Medical School, Department of Neurology, Hannover, Germany; Carl von Ossietzky University of Oldenburg, Department for Health Services Research, Oldenburg, Germany; University Medical Center Hamburg-Eppendorf, Department of Medical Psychology, Hamburg, Germany; Carl von Ossietzky University of Oldenburg, Department for Health Services Research, Oldenburg, Germany; MS Research and Project Development gGmbH (MSFP), German MS-Registry, Hannover, Germany; Neurological Rehabilitation Center Quellenhof, 39555 Sana Kliniken Bad Wildbad GmbH, Bad Wildbad, Germany; Deutsche Multiple Sklerose Gesellschaft (DMSG), Landesverband Niedersachsen, Hannover, Germany; AOK Niedersachsen, Health Services Research Unit, Hannover, Germany; Carl von Ossietzky University of Oldenburg, Department of Special Needs Education and Rehabilitation, Oldenburg, Germany; Hannover Medical School, Institute for Epidemiology, Social Medicine and Health Systems Research, Hannover, Germany; Center for Health Economics Research Hannover (CHERH), Hannover, Germany; MS Research and Project Development gGmbH (MSFP), German MS-Registry, Hannover, Germany; AOK Niedersachsen, Health Services Research Unit, Hannover, Germany; Carl von Ossietzky University of Oldenburg, Department of Special Needs Education and Rehabilitation, Oldenburg, Germany; University of Rostock, Department of Neurology, Division of Neuroimmunology, Rostock, Germany; Deutsche Multiple Sklerose Gesellschaft (DMSG), Landesverband Niedersachsen, Hannover, Germany; DIAKOVERE Henrietten Hospital, Department of Neurology and Clinical Neurophysiology, Hannover, Germany; Hannover Medical School, Institute for Epidemiology, Social Medicine and Health Systems Research, Hannover, Germany; Center for Health Economics Research Hannover (CHERH), Hannover, Germany

**Keywords:** Multiple sclerosis, health services administration, insurance claim review, health care surveys, data linkage, cluster analysis

## Abstract

**Background:**

Previous investigations of multiple sclerosis (MS)-related healthcare have focused on utilisation of specific individual health services (e.g. hospital care, office-based neurologists) by people with MS (PwMS). Meanwhile, little is known about possible patterns of utilisation across health services and their potential differences across patient characteristics.

**Objective:**

To comprehensively analyse and identify patterns of MS-related health service utilisation and detect patient characteristics explaining such patterns.

**Methods:**

In 2021, we invited all PwMS insured by the largest insurance company in Lower Saxony, Germany, to take part in an online survey. We merged respondents’ survey and health insurance claims data. We analysed MS-related health service utilisation and defined individual characteristics for subgroup analyses based on Andersen's Behavioural Model. We executed non-parametric missing value imputation and conducted hierarchical clustering to find patterns in health service utilisation.

**Results:**

Of 6928 PwMS, 1935 responded to our survey and 1803 were included in the cluster analysis. We identified four distinct health service utilisation clusters: (1) regular users (n = 1130), (2) assistive care users (n = 443), (3) low users (n = 195) and (4) special services users (n = 35). Clusters differ by patient characteristics (e.g. age, impairment).

**Conclusion:**

Our findings highlight the complexity of MS-related health service utilisation and provide relevant stakeholders with information allowing them to tailor healthcare planning according to utilisation patterns.

## Introduction

The prevalence of people with multiple sclerosis (PwMS) has increased in recent years to about 2.8 million worldwide,^1^ and more than 240,000 in Germany.^2^ Depending on MS course, disease activity, symptoms, and individual preferences, MS therapy has become highly sophisticated,^3^ leading to presumably varying utilisation patterns. Despite this, research on MS-related health service utilisation has predominantly focused on individual health services (e.g. hospital care, office-based neurologists).^2,4–6^

The German healthcare system is organised into sectors (inpatient and outpatient services). To the extent that this is feasible, routine care is provided within the outpatient sector, while inpatient care is ideally reserved for more urgent or complex matters. Outpatient physicians provide specific services within the scope of their specialisation (e.g. office-based neurologists can opt to prescribe immunomodulatory therapy). The choice of service provider is generally left to the patient, but may be limited by a shortage of capacity at the desired provider.^7^ More specialised care is usually initiated through referral by outpatient physicians (e.g. outpatient hospital-based services are provided for more complex disease characteristics upon referral). The complex patterns of provision across MS-related health services are not yet comprehensively understood.^8^ Therefore, a mixed-methods study *“Multiple Sclerosis–Patient-Oriented Care in Lower Saxony” (MS-PoV)*,^9^ was conducted in the federal state of Lower Saxony, Germany to examine health service utilisation and identify influencing factors.

We adopted Andersen's Behavioural Model as a theoretical framework for this analysis. This social behavioural model is a well-established framework to structure and analyse determinants of health behaviours and outcomes,^10,11^ which has been implemented in various healthcare and disease contexts,^12^ including MS-specific research.^4,13^ Contextual and individual predisposing, enabling and need factors are potential explanatory variables for health behaviours (e.g. personal health service utilisation) and outcomes (e.g. quality of life). While predisposing characteristics refer to demographic, social and mental aspects, enabling characteristics comprise financing and organisational factors. Need characteristics include perceived and evaluated need factors driving health service utilisation.^10–12^ Focussing on individual characteristics to evaluate the study population, we address the following questions: (1) What health services do PwMS use?, (2) Can patterns of health service utilisation be identified? and (3) How do PwMS differ depending on the clusters to which they belong?

## Materials and methods

For a general overview of the entire study methodology, please refer to the published study protocol^9^ and registration at German Clinical Trials Register DRKS00021741. Approval was obtained by the Ethic Committees of the Hannover Medical School (9173_BO_K_2020) and of the University of Oldenburg (2020–108)*.* In the current section, we describe in detail the core element of the study project's quantitative analyses: the merging of primary (online survey) and secondary (health insurance claims) data.

### Study population

Individuals insured by AOK Lower Saxony (the largest statutory health insurance company in Lower Saxony with a market share of almost 40%^14^) who were at least 18 years of age, living in Lower Saxony and diagnosed with either course of MS (ICD-10 G35.-) were included in the analyses. Insured persons were eligible if they fulfilled any of the following criteria in 2019 or 2020: (1) at least two MS diagnoses in two different quarters in the outpatient setting, (2) at least one outpatient MS diagnosis accompanied by immunomodulatory MS therapy or (3) at least one outpatient MS diagnosis accompanied by at least one inpatient MS diagnosis.

### Data sources

#### Online survey

A broad literature search and focus group discussions with PwMS and neurologists were conducted to develop a comprehensive online survey allowing us to capture information on health service utilisation and patient-reported outcomes. The online survey was iteratively pretested by persons with (n = 9) and without MS (n = 38) to identify both disease-specific and general challenges of clarity and subsequently adapted. Respondents could pause the online survey as needed and continue as long as the platform (LimeSurvey Version 3.28.74^15^) was active. A telephone hotline was available to all invitees for any inquiries regarding the survey. To keep the questionnaire as short as possible for each participating individual, branching logic was implemented; thus, more detailed questions were only presented to those for whom they were relevant (‘missing by design’). As an incentive, the participants were asked at the end of the survey whether they wanted to take part in a prize draw for a fully organised visit to the local zoo with their families. Individualised postal invitation letters were sent to all eligible persons in September 2021. Participation status was monitored and up to two postal reminders were sent to those who had not yet participated after 2 and 4 weeks. The cut-off date for acquisition was 31 October 2021.

#### Insured persons’ health insurance claims data

Health insurance claims data is generated for billing purposes and contains information on services in the healthcare sector and associated diagnoses.^16^ For PwMS who participated in the online survey, survey data was linked with outpatient and inpatient claims data (Table A1) using pseudonymised study identification codes, based on digitally obtained informed consent.

### Variables

**Figure 1.**
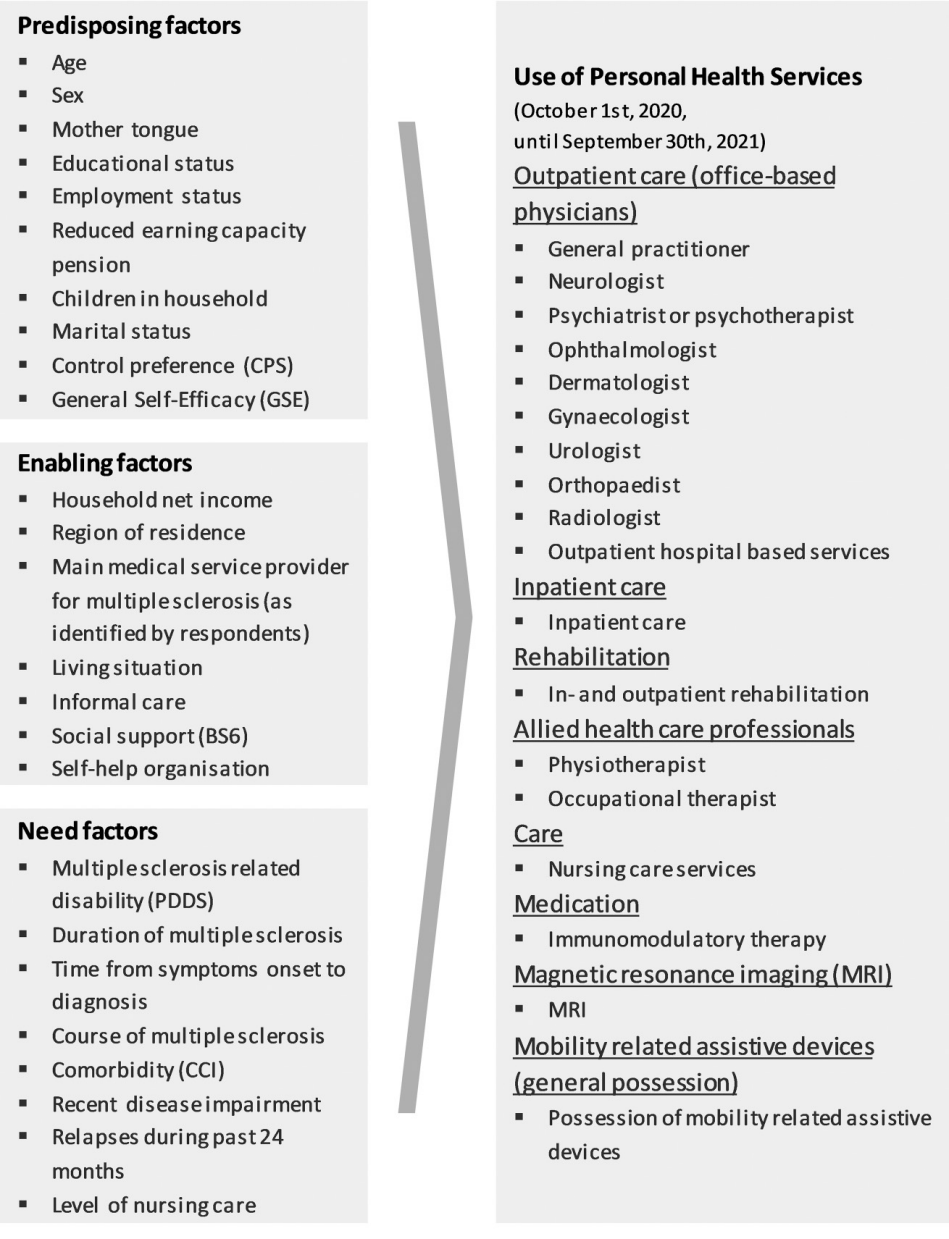
Identified variables in the framework of Andersen's Behavioural Model.^11^

Variable selection was guided by Andersen's Behavioural Model (Figure 1).^11^ For PwMS included in the analysis, we depicted MS-associated utilisation of healthcare services from 1 October 2020 until 30 September 2021 by selecting healthcare services in their claims data, coded with a MS diagnosis. Some healthcare services can be entered into claims data without an associated diagnosis; these were included by default. To describe individual characteristics of PwMS, we included standardised and self-developed complementary items from the survey (e.g. Control Preference Scale [CPS],^17^ General Self-Efficacy [GSE] scale,^18^ Brief Social Support [BS6] scale,^19^ Patient Determined Disease Steps [PDDS] scale^20^) and claims data (e.g. Charlson Comorbidity Index [CCI]^21^). For a comprehensive item definition (online survey and health insurance claims data), please refer to the supplementary material (Table A1).

### Data analyses

Data preparation and analyses were conducted using SPSS (28.0.1.0) and RStudio (2022.07.2) in R (4.2.3).^22^ Data sets were prepared for analyses (analysis of distribution of missing data, plausibility checks etc.). According to Newman,^23^ all partial and full respondents should be included in analyses. Therefore, we conducted descriptive analyses in the online survey data set excluding all PwMS that did not answer a single question. For further analyses, we reduced the online survey data set and excluded all participants with more than 25% missing values in their entire survey. Due to the structure of the data, non-parametric missing value imputation was conducted for the remaining participants with missing values,^24^ which performed well (normalised root mean squared error [NRMSE] and proportion of falsely classified entries [PFC] close to 0). For sensitivity analyses, we analysed the online survey data set without imputations. Standardised scales were aggregated accordingly (Table A1). We conducted non-responder analyses including all *individual characteristics* defined in the claims data set (Table A2).

The study population is described by *individual characteristics (predisposing, enabling, need factors)*. For cluster analyses, we grouped *utilisation of personal health services* as outcome variables inspired by Beckerman et al.,^4^ our findings of frequently used health services, and expert discussions (CT, FH, PF, UKZ). Variables included in cluster analyses were binary coded (utilisation vs. non-utilisation) as suggested by Recchia et al.^25^ Various methods for cluster analyses exist and have previously been used in health services research.^25–27^ As clustering method may directly affect clustering results, we tested various clustering methods (model-based, partitioning, hierarchical) on our data. We chose a hierarchical clustering approach as this method yielded the most reasonable results and does not require a priori determination of the optimal number of clusters for cluster identification. We chose the clustering algorithm *hclust* from the R *stats* package,^22^ based on the Gower dissimilarity measure derived with the *daisy* function from the R *cluster* package.^28^ Based on the dissimilarity measure, individuals are grouped together according to chosen linkage measures. As proposed by van Allen et al.,^29^ we tested various linkage methods (complete, single, average, centroid, Ward) (Figures A1–A5). Only complete- and Ward-linkage yielded reasonable and interpretable group sizes (Figures A1 and A5). As previously shown in the literature,^27,30^ clustering metrics might not reliably yield reasonable clustering results. Based on the dendrograms (Figure A1 and A5), where larger heights indicate greater differences in the process of linking groups, two-cluster solutions would have been reasonable (Figures A6 and A7). After manual review of potential clusters, we opted for a four-cluster solution in order to discover a greater variety of utilisation patterns. The complete linkage method led to significant differences for all included services (Table A3). The final linkage method and optimal number of clusters were therefore chosen according to the dendrogram (Figure A1) and content-wise interpretation. Identified clusters are compared descriptively, focussing on individual characteristics of enclosed PwMS. Fisher's exact test from the R *stats* package^22^ was used to analyse statistically significant relationships and Cramer's V from the R *rcompanion* package^31^ to identify corresponding effect sizes. The Strengthening the Reporting of Observational Studies in Epidemiology (STROBE) statement^32^ was adhered to.

### Data availability

The data sets analysed were used under licence for the current study. The open distribution of the data is therefore prohibited by the data protection regulations effective in Lower Saxony. The data remained on a limited access secure research environment throughout analyses. For legal and ethical reasons, the data cannot leave this secure research environment. To find individual solutions, researchers may contact the authors upon reasonable request.

## Results

**Figure 2.**
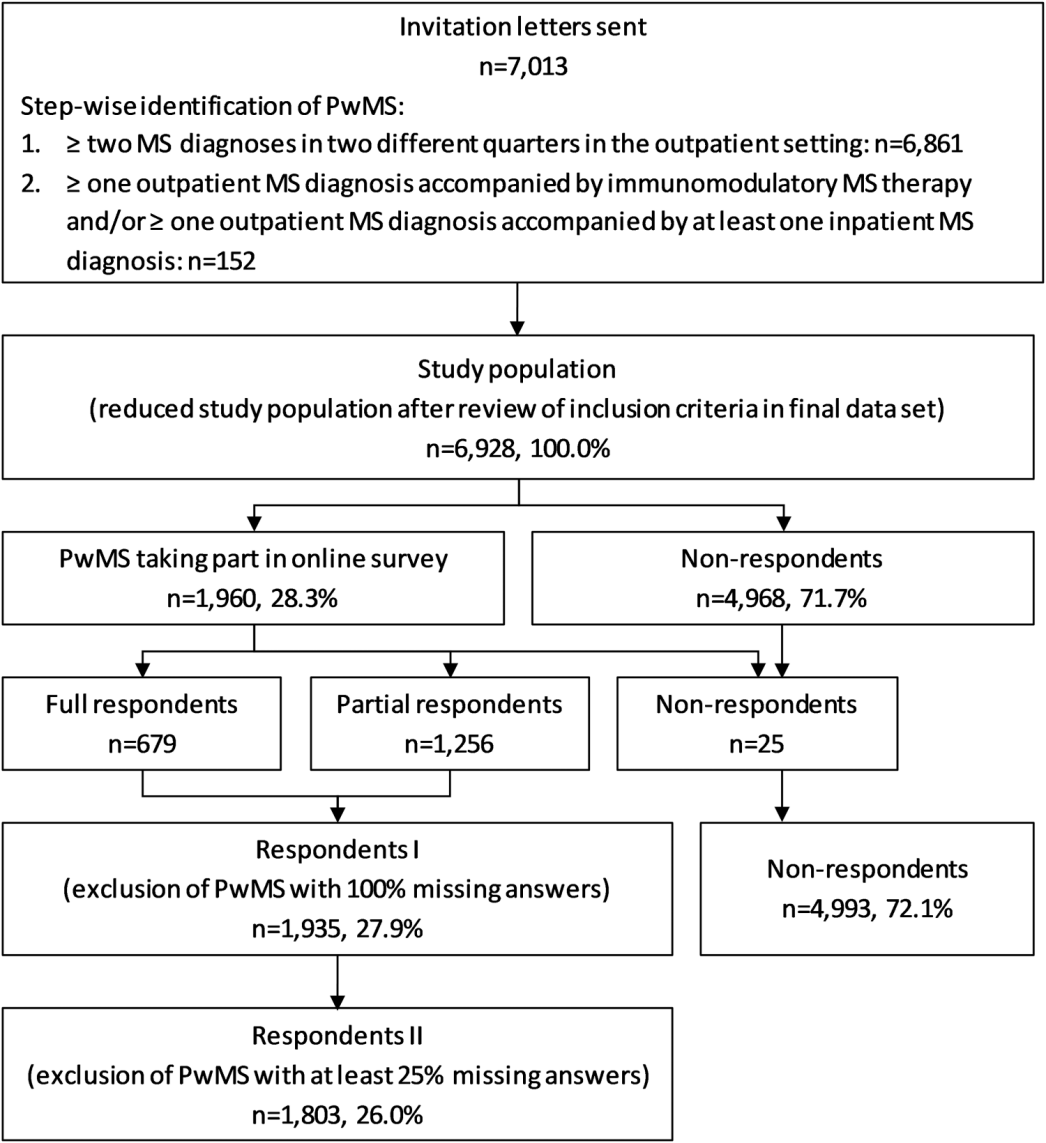
Flow chart of study participants. Number of patients (n); people with multiple sclerosis (PwMS).

The final study population comprises 6928 PwMS meeting inclusion criteria. A merging of online survey data and claims data was conducted for a total of 1935 (27.9%) PwMS who took part in the online survey (Respondents I). Of those, 132 participants were missing more than 25% of the answers in the survey and were excluded from subsequent statistical analyses (Respondents II) (Figure 2). The non-responder analyses showed significant differences between respondents and all invited PwMS regarding age and, subsequently, general impairment and health care utilisation variables. As those effects were rather small, we only report them here (Table A2).

*Individual characteristics* of the study population were structured according to the framework of Andersen's Behavioural Model^11^ into *predisposing*, *enabling* and *need* factors (Figure 1, Table 1).

**Table 1.** Study population (Respondents I).

Variables within Andersen's behavioural model		n	%
Sample		1935	100.0
**Individual characteristics**			
***Predisposing***			
Age (mean 49.5 years, SD 12.2 years)			
	18–35 years	293	15.1
	36–50 years	661	34.2
	51–65 years	820	42.4
	66 years and older	161	8.3
Sex			
	Female	1374	71.0
	Male	561	29.0
Mother tongue^a^			
	German	1639	91.8
	Other	146	8.2
Educational status^a^			
	Low	483	27.3
	Moderate	1039	58.7
	High	141	8.0
	Other	106	6.0
Employment status^a^			
	Employed	930	52.2
	Not employed	853	47.8
Reduced earning capacity pension^a^			
	Without reduced earning capacity pension	1135	63.9
	With reduced earning capacity pension	642	36.1
Children in household^a^			
	None	1390	79.5
	One	178	10.2
	Two or more	180	10.3
Marital status^a^			
	Serious relationship	1397	78.0
	Non-serious relationship	395	22.0
Control preference (CPS)^a^			
	Active	719	40.4
	Collaborative	868	48.8
	Passive	191	10.7
General self-efficacy (GSE)^a^			
	Low self-efficacious	923	54.0
	High self-efficacious	787	46.0
***Enabling***			
Household net income^a^			
	Risk of poverty	699	42.6
	No risk of poverty	942	57.4
Region of residence			
	Urban	393	20.3
	Suburban	655	33.9
	Rural	887	45.8
Main medical service provider for multiple sclerosis (self-identified)^a^			
	Outpatient general practitioner	126	6.8
	Outpatient neurologist	1443	77.7
	Specialised MS centre	203	10.9
	Other	13	0.7
	None	73	3.9
Living situation^a^			
	Alone	321	17.8
	With family or spouse	1421	78.9
	Other	58	3.2
Informal care^a^			
	No support received	1037	58.4
	Support received	738	41.6
Social support (BS6)^a^			
	Low	245	13.8
	Moderate	475	26.8
	High	695	39.2
	Very high	360	20.3
Self-help organisation^a^			
	Member	291	15.9
	No member	1538	84.1
***Need***			
Multiple sclerosis related disability (PDDS)^a^			
	Mild (score 0–2)	983	55.2
	Moderate (score 3–5)	543	30.5
	Severe (score 6–8)	254	14.3
Duration of multiple sclerosis^a^			
	Less than 2 years	87	4.8
	2–15 years	986	54.5
	More than 15 years	707	39.1
	Unknown	29	1.6
Time from symptoms onset to diagnosis^a^			
	Up to 1 year	896	49.6
	2–6 years	441	24.4
	More than 6 years	312	17.3
	After diagnosis	34	1.9
	Unknown	122	6.8
Course of multiple sclerosis^a^			
	Relapsing	1040	57.6
	Non-relapsing	470	26.0
	Unknown	296	16.4
Comorbidity (CCI index)			
	None	970	50.1
	1–2	640	33.1
	3–4	243	12.6
	5 or more	82	4.2
Recent disease impairment^a^			
	More	1030	59.4
	Same	623	35.9
	Less	81	4.7
Relapses during past 24 months^a^			
	None	913	51.0
	One or more	476	26.6
	Unknown	402	22.5
Level of nursing care			
	No or little impairment	1514	78.2
	Significant impairment	421	21.8

Abbreviations: BS6: Brief Social Support scale; CCI: Charlson Comorbidity index; CPS: Control Preference Scale; GSE: General Self-Efficacy Scale; MS: multiple sclerosis; n: number of patients; PDDS: patient determined disease steps, SD: standard deviation. No missing values present in variables solely based on health insurance claims data due to completeness of data source in this analysis.

^a^ Denominators may differ due to missing values.

MS-related health service utilisation was analysed by individual health services across sectors (Table 2). In the outpatient sector, most PwMS consult office-based general practitioners (GP) (n = 1,789, 92.5%) and/or neurologists (n = 1,652, 85.4%). Utilisation of psychiatrists or psychotherapists was coded for fewer respondents (n = 100, 5.2%), while 41.6% (n = 805) visit physiotherapists. More than a fifth of the study population uses nursing care services (n = 417, 21.6%) and 26.6% own a mobility-related assistive device (n = 515). Immunomodulatory therapy was used by 60.6% (n = 1173) of the participating PwMS, and almost 40% (n = 754) have had a magnetic resonance imaging (MRI) scan. Exclusively inpatient services, such as hospitalisation (n = 165, 8.5%), were less commonly utilised.

**Table 2.** Utilisation of personal health services during a 12 months period (Respondents I).

Variables within Andersen's Behavioural Model	n	%	Mean (SD) among PwMS utilising health service^20^
Sample	1935	100.0	
		
Outpatient care (office-based physicians)	*Health service utilised (yes)*		*Number of contacts per study participant on which services were charged for*
General practitioner	1789	92.5	9.78 (7.48)
Neurologist	1652	85.4	7.28 (4.62)
Psychiatrist or psychotherapist	100	5.2	5.24 (6.66)
Ophthalmologist	422	21.8	1.84 (1.67)
Dermatologist	113	5.8	2.10 (2.72)
Gynaecologist	527	27.2	2.48 (2.51)
Urologist	268	13.9	4.91 (4.24)
Orthopaedist	254	13.1	3.04 (3.17)
Radiologist	703	36.3	1.47 (0.83)
		
	*Health service utilised (yes)*		*Count of days per study participant on which services were initialised*
Outpatient hospital based services	175	9.0	2.19 (1.41)
		
Inpatient care	*Health service utilised (yes)*		*Count of hospitalised days per study participant*
Inpatient care	165	8.5	11.61 (23.99)
		
Rehabilitation	*Health service utilised (yes)*		*Count of days within timeframe of rehabilitation services per study participant*
In- and outpatient rehabilitation	84	4.3	26.24 (10.20)
		
Allied healthcare professionals	*Health service utilised (yes)*		*Count of primary service units per study participant on which services were charged*
Physiotherapist	805	41.6	49.88 (34.65)
Occupational therapist	179	9.3	38.51 (26.53)
		
Care	*Health service utilised (yes)*		
Nursing care services	417	21.6	NA
		
Medication	*Health service utilised (yes)*		
Immunomodulatory therapy	1173	60.6	NA
		
MRI scan	*Health service utilised (yes)*		
MRI	754	39.0	NA
		
Mobility related assistive devices	*Health service utilised (yes)*		
Possession of mobility related assistive devices^a^	515	26.6	NA

Abbreviations: MRI: magnetic resonance imaging; n: number of patients; NA: not available; PwMS: people with multiple sclerosis; SD: standard deviation.

^a^ Online survey: further possible answers: no (n = 1,316, 68.0%) or missing (n = 104, 5.4%).

**Figure 3.**
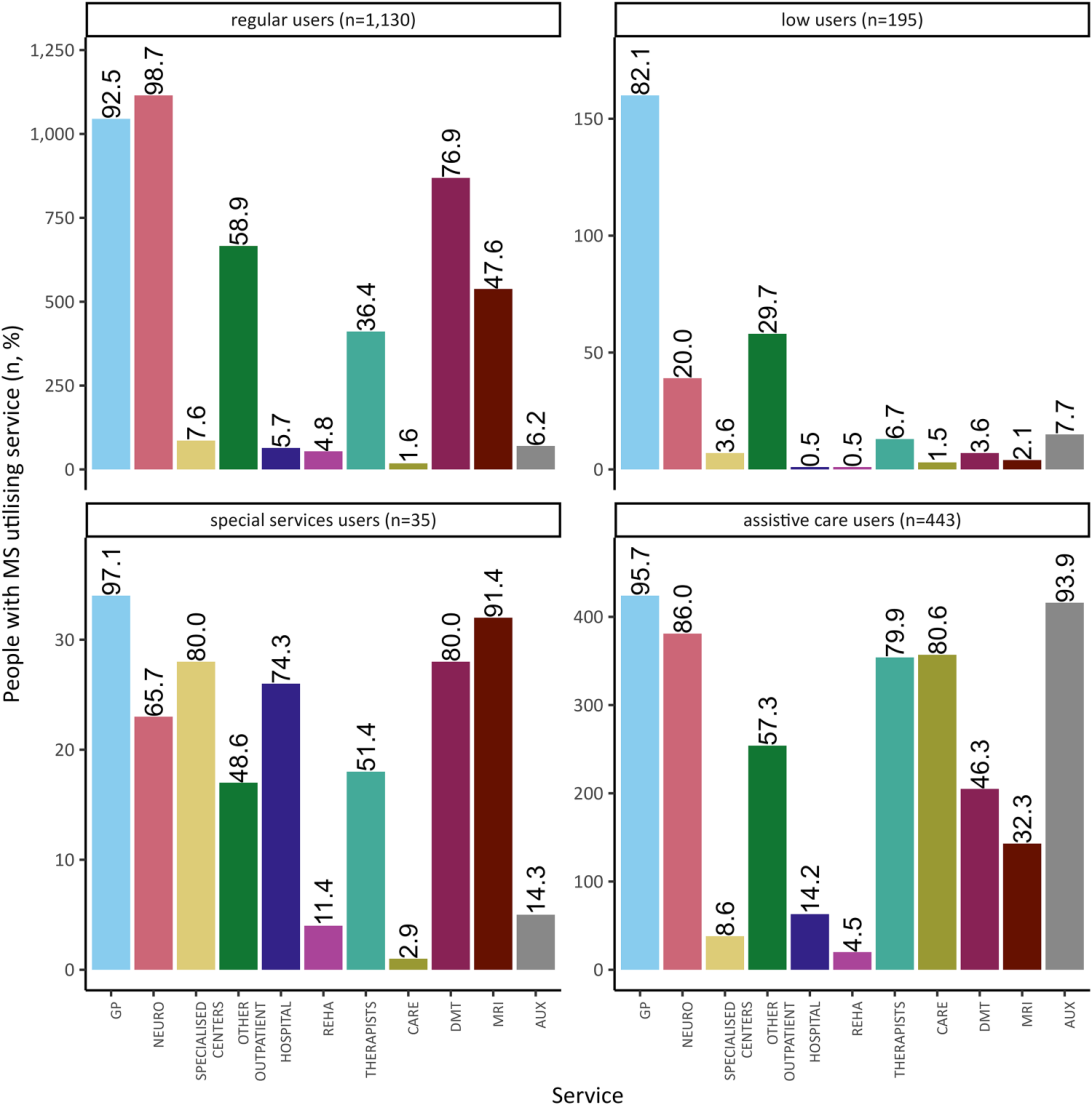
Identified clusters of health service utilisation (Respondents II). Office-based general practitioner (GP); office-based neurologist (NEURO); outpatient hospital based services (SPECIALISED CENTERS); office-based ophthalmologist, gynaecologist, urologist orthopaedist (OTHER OUTPATIENT); inpatient care (HOSPITAL); in- and outpatient rehabilitation (REHA); physiotherapist, occupational therapist (THERAPISTS); nursing care services (CARE); immunomodulatory therapy (DMT); magnetic resonance imaging (MRI); possession of mobility related assistive devices (AUX).

We identified four distinct clusters (p<0.05, Figure 3, Table A3): (1) regular users (n = 1130), (2) assistive care users (n = 443), (3) low users (n = 195) and (4) special services users (n = 35).

Regarding the effect size, the greatest differentiation in health service utilisation between clusters is defined by the number of encounters with office-based neurologists (Cramer's V = 0.703) and, physiotherapists and occupational therapists (Cramer's V = 0.453), along with use of nursing care services (Cramer's V = 0.834) or, immunomodulatory therapy (Cramer's V = 0.493), and possession of mobility-related assistive devices (Cramer's V = 0.837) (Table A3). Each cluster has a relatively high percentage of PwMS who have visited an office-based GP (Figure 3). PwMS enclosed in the cluster *regular users* predominantly consult outpatient neurologists for their MS (98.7%), while immunomodulatory therapy (76.9%) and MRI (47.6%) are quite common. Most *low users* predominantly consult an outpatient GP (82.1%), but other services are utilised far less than in other clusters. The cluster of *special services users* is characterised by a high percentage of PwMS visiting outpatient hospital based services (80.0%), hospitals (74.3%) and utilising MRI scans (91.4%). The amount of PwMS on immunomodulatory therapy is comparatively high (80.0%) in this cluster. Meanwhile, many PwMS in the last cluster, *assistive care users,* visited outpatient therapists (e.g. physiotherapists) (79.9%) and are in need of basic nursing care provision (80.6%), while almost all possess mobility-related assistive devices (93.9%).

The characteristics of PwMS differ between clusters. A detailed overview is shown in Table 3. For sensitivity analyses, please refer to Table A4.

**Table 3.** Subgroup analyses of identified clusters of health service utilisation (Respondents II).

Cluster	Regular users (n = 1130)		Assistive care users (n = 443)		Low users (n = 195)		Special services users (n = 35)		Fisher's exact test	Cramer's V^a^
Variables within Andersen's Behavioural Model	N	%	N	%	N	%	N	%	p-value	Effect size
**Predisposing**										
Age (years)	Mean 46.6 SD 11.4		Mean 56.6 SD 10.6		Mean 51.0 SD 12.9		Mean 42.7 SD 12.4			
									<0.001*	0.201
18–35	217	19.2	14	3.2	30	15.4	*13*	*37.1*		
36–50	*454*	*40.2*	103	23.3	54	27.7	12	34.3		
51–65	421	37.3	*244*	*55.1*	85	43.6	9	25.7		
65 and older	38	3.4	*82*	*18.5*	26	13.3	1	2.9		
Sex									0.1	0.054
Female	*822*	*72.7*	304	68.6	135	69.2	21	60.0		
Male	308	27.3	139	31.4	60	30.8	*14*	*40.0*		
Mother tongue									0.5	0.036
German	1039	91.9	*411*	*92.8*	178	91.3	30	85.7		
Other	91	8.1	32	7.2	17	8.7	*5*	*14.3*		
Educational status									<0.001*	0.118
Low	249	22.0	*188*	*42.4*	63	32.3	7	20.0		
Moderate	*718*	*63.5*	199	44.9	110	56.4	22	62.9		
High	101	8.9	26	5.9	12	6.2	*4*	*11.4*		
Other	62	5.5	*30*	*6.8*	10	5.1	2	5.7		
Employment status									<0.001	0.490
Employed	*766*	*67.8*	43	9.7	110	56.4	20	57.1		
Not employed	364	32.2	*400*	*90.3*	85	43.6	15	42.9		
Reduced earning capacity pension									<0.001	0.392
Without reduced earning capacity pension	835	73.9	137	30.9	*151*	*77.4*	28	0.8		
With reduced earning capacity pension	295	26.1	*306*	*69.1*	44	22.6	7	0.2		
Children in household									<0.001*	0.130
None	843	74.7	*401*	*91.8*	152	78.4	25	73.5		
One	149	13.2	18	4.1	19	9.8	*7*	*20.6*		
Two or more	*137*	*12.1*	18	4.1	23	11.9	2	5.9		
Marital status									<0.001	0.122
Serious relationship	*925*	*81.9*	321	72.5	135	69.2	26	74.3		
Non-serious relationship	205	18.1	122	27.5	*60*	*30.8*	9	25.7		
Control preference (CPS)									<0.001*	0.102
Active	453	40.3	145	34.0	*82*	*55.8*	17	50.0		
Collaborative	*566*	*50.4*	210	49.3	54	36.7	14	41.2		
Passive	105	9.3	*71*	*16.7*	11	7.5	3	8.8		
General self-efficacy (GSE)									<0.001	0.226
Low self-efficacious	551	48.8	*327*	*73.8*	85	43.6	16	45.7		
High self-efficacious	579	51.2	116	26.2	*110*	*56.4*	19	54.3		
**Enabling**										
Household net income									<0.001	0.098
Risk of poverty	435	38.5	*238*	*54.5*	93	47.9	17	50.0		
No risk of poverty	*694*	*61.5*	199	45.5	101	52.1	17	50.0		
Region of residence									0.1*	0.054
Urban	230	20.4	89	20.1	*43*	*22.1*	4	11.4		
Suburban	383	33.9	*158*	*35.7*	62	31.8	6	17.1		
Rural	517	45.8	196	44.2	90	46.2	*25*	*71.4*		
Main medical service provider for multiple sclerosis (self-identified)									<0.001*	0.289
General practitioner	25	2.2	46	10.4	*43*	*22.1*	2	5.7		
Outpatient neurologist	*975*	*86.3*	328	74.0	88	45.1	15	42.9		
Specialised MS centre	118	10.4	50	11.3	13	6.7	*17*	*48.6*		
Other	6	0.5	2	0.5	*3*	*1.5*	0	0.0		
None	6	0.5	17	3.8	*48*	*24.6*	1	2.9		
Living situation									<0.001*	0.100
Alone	166	14.7	95	21.4	*52*	*26.7*	7	20.0		
With family or spouse	*937*	*82.9*	325	73.4	139	71.3	25	71.4		
Other	27	2.4	23	5.2	4	2.1	*3*	*8.6*		
Informal care									<0.001	0.537
No support received	812	71.9	51	11.5	*155*	*79.5*	23	65.7		
Support received	318	28.1	*392*	*88.5*	40	20.5	12	34.3		
Social support (BS6)									<0.001*	0.085
Low	160	14.2	37	8.4	*44*	*22.6*	5	14.3		
Moderate	286	25.3	122	27.5	59	30.3	*17*	*48.6*		
High	454	40.2	*190*	*42.9*	59	30.3	9	25.7		
Very high	230	20.4	*94*	*21.2*	33	16.9	4	11.4		
Self-help organisation									<0.001	0.137
Member	153	13.5	*109*	*24.6*	21	10.8	5	14.3		
No member	977	86.5	334	75.4	*174*	*89.2*	30	85.7		
**Need**										
Multiple sclerosis-related disability (PDDS)									<0.001*	0.488
Mild	*810*	*71.7*	34	7.7	134	68.7	20	57.1		
Moderate	302	26.7	*179*	*40.4*	56	28.7	14	40.0		
Severe	18	1.6	*230*	*51.9*	5	2.6	1	2.9		
Duration of multiple sclerosis									<0.001*	0.173
Less than 2 years	72	6.4	5	1.1	4	2.1	*6*	*17.1*		
2–15 years	*698*	*61.8*	155	35.0	105	53.8	19	54.3		
More than 15 years	341	30.2	*277*	*62.5*	82	42.1	10	28.6		
Unknown	19	1.7	6	1.4	*4*	*2.1*	0	0.0		
Time from symptoms onset to diagnosis									<0.001*	0.081
Up to 1 year	595	52.7	170	38.4	*104*	*53.3*	14	40.0		
2–6 years	263	23.3	124	28.0	44	22.6	*10*	*28.6*		
More than 6 years	174	15.4	105	23.7	31	15.9	*9*	*25.7*		
After diagnosis	21	1.9	*13*	*2.9*	3	1.5	*1*	*2.9*		
Unknown	77	6.8	*31*	*7.0*	13	6.7	1	2.9		
Course of multiple sclerosis									<0.001*	0.196
Relapsing	718	63.5	186	42.0	114	58.5	*23*	*65.7*		
Non-relapsing	207	18.3	*208*	*47.0*	46	23.6	7	20.0		
Unknown	*205*	*18.1*	49	11.1	35	17.9	5	14.3		
Comorbidity (CCI index)									<0.001*	0.205
None	*675*	*59.7*	99	22.3	116	59.5	18	51.4		
1–2	333	29.5	194	43.8	53	27.2	*16*	*45.7*		
3–4	98	8.7	*108*	*24.4*	18	9.2	1	2.9		
5 or more	24	2.1	*42*	*9.5*	8	4.1	0	0.0		
Recent disease impairment									<0.001*	0.212
More	550	48.7	*368*	*83.1*	94	48.2	19	54.3		
Same	520	46.0	66	14.9	*92*	*47.2*	14	40.0		
Less	60	5.3	9	2.0	9	4.6	*2*	*5.7*		
Relapses during past 24 months									<0.001	0.106
None	*599*	*53.0*	197	44.5	103	52.8	8	22.9		
One or more	531	47.0	246	55.5	92	47.2	*27*	*77.1*		
Level of nursing care									<0.001	0.834
No or little impairment	*1111*	*98.3*	84	19.0	191	97.9	34	97.1		
Significant impairment	19	1.7	*359*	*81.0*	4	2.1	1	2.9		

Abbreviations: BS6: Brief Social Support scale; CCI: Charlson Comorbidity index; CPS: Control Preference Scale; GSE: General Self-Efficacy Scale; n: number of patients; PDDS: Patient Determined Disease Steps; SD: standard deviation. *Highest value in comparison to variable characteristics in other clusters.*

^a^ Effect size: small 0.06 to <0.17, moderate: 0.17 to <0.29, large: ≥0.29.

* Monte Carlo simulated p-value with 100,000 replicates

Focussing on *predisposing* factors, PwMS in the largest cluster, *regular users,* are relatively younger (mean 46.5 years), moderately educated (63.5%), and more often employed (67.8%) compared to other cluster populations. The majority is in a serious relationship (81.9%) and more PwMS in this cluster live with children (25.3%). Meanwhile, PwMS in the cluster *low users* are relatively older (mean 51.0 years), their educational status is lower (low: 32.3%, moderate: 56.4%), fewer are employed (56.4%) and the percentage not in a serious relationship is highest (30.8%). In the cluster *special services users,* PwMS are predominately younger (mean 42.7 years) and the proportion of PwMS with a high level of education is greater (high: 11.4%). In the second-largest cluster, *assistive care users*, PwMS are comparatively older (mean 56.6 years) and most are not employed (90.3%), nor do they live with children (91.8%). The educational status is lower (low: 42.4%) and many PwMS define their self-efficacy as low (73.8%).

Regarding *enabling* factors, the majority of PwMS in the cluster *regular users* lives with their family or spouse (82.9%). In this cluster, the main medical service provider for MS identified by respondents is overwhelmingly the outpatient neurologist (86.3%). In contrast, PwMS in the cluster *low users* identify their outpatient GP (22.1%), none (24.6%), or their outpatient neurologist (45.1%) as their main medical service provider for MS. A larger proportion of PwMS in this cluster lives alone (26.7%). The cluster *special services users* is defined by a relatively high percentage of PwMS referring to specialised MS centres (such as outpatient departments at hospitals) as their main medical service provider (48.6%), though many in this cluster name their office-based neurologist as well (42.9%). More than half of PwMS in the cluster *assistive care users* are under risk of poverty (54.5%) and 74.0% refer to the outpatient neurologist as their main medical service provider. Noticeable differences can also be seen for the amount of support received, which is highest in this cluster (informal care: 88.5%, social support high or very high: 64.1%).

Concentrating on *need* factors, 63.5% of PwMS in the cluster *regular users* self-identify a relapsing MS course. Compared to other clusters, the amount of PwMS who do not know their MS course is higher (18.1%). Those in this cluster predominantly report mild MS-related disability (PDDS) (71.1%) and moderate MS duration (2–15 years: 61.8%). In comparison, fewer PwMS in the cluster *low users* report moderate duration (2-15 years: 53.8%). Additionally, the percentage of non-relapsing MS is higher (23.6%) in this cluster, while that of relapsing MS is lower (58.5%) and disease duration is more often longer (more than 15 years: 42.1%). The amount of recent diagnoses (obtained less than 2 years ago) is highest (17.1%) in the cluster *special services users*. Two thirds of PwMS in this cluster identify their MS course as relapsing (65.7%) and even more report one or more relapses during the past 24 months (77.1%), while relapses within this period are reported by only about half of PwMS in other clusters. In the cluster *assistive care users,* 62.5% of PwMS have had a MS diagnosis for more than 15 years. Compared with other clusters, more PwMS in this cluster identify their MS course as non-relapsing (47.0%). A noticeably high proportion of PwMS reports disease impairment over the past 24 months (83.1%), and an overall significant level of impairment (81.0%) was identified.

## Discussion

To the best of our knowledge, this is the first study to identify patterns of MS-related health service utilisation across services and sectors. Cluster analysis is explorative in nature and other cluster solutions could exist. Nonetheless, our results have been proven to offer a reliable solution, as frequencies of utilisation of included health services differ significantly across clusters. We identified a cluster of comparatively young (mean 42.7 years) and more frequently recently diagnosed PwMS (17.1%) using predominantly specialised services (*special services users*). As PwMS in this cluster refer to specialised MS centres (self-identified) as their main medical service provider and their MS-related disability is mostly mild to moderate (PDDS), we assume PwMS with recent diagnoses and/or more complex MS courses are enclosed. PwMS with a mean age of 46.5 years and the greatest proportion of moderate disease duration (61.8%) are enclosed in the largest cluster, *regular users*. PwMS in this utilise neurological services, but a smaller number of specialised health services (e.g. outpatient hospital based services), while most report mild MS related disability. The cluster *assistive care users* is defined by the most widespread utilisation of auxiliary services (e.g. nursing care and mobility-related assistive devices). PwMS in this cluster are on average 56.6 years old. Most have had MS for more than 15 years (62.5%), and self-identify comparatively high levels of MS-related disability. The cluster in which PwMS only utilise a small number of services is noteworthy (*low users*). Interestingly, this cluster includes the largest number of PwMS with steady disease impairment and independent lifestyles, with those in this cluster being older (mean 51.0 years) and less educated than *regular users*. Non-responder analyses revealed the comparatively younger age of the analysed study population (respondents vs. entire sample). In analyses based on other samples, we would expect similarly characterised clusters comprising a relatively differing number of included PwMS each (e.g. a relatively larger cluster of *assistive care users*), as the regulation of access to care and reimbursement of health services in the statutory health insurance system is largely uniform in Germany.^7^ Further analyses would be useful to confirm these assumptions, but cannot be performed with our present data as information concerning mobility-related assisted devices is relevant for clustering and in the chosen definition available only for survey respondents.

Strengths of our study are the linkage of data sources (survey and claims data) and the structural guidance by a well-established framework.^11^ Nonetheless, inconsistencies in the categorisation of characteristics that supposedly influence health service utilisation have previously been shown,^12^ and variables possibly relevant to describing utilisation clusters could be missing (e.g. smoking^33^). Further limitations of our study predominantly arise from our data sources. For example, survey data is prone to selection or recall bias,^34^ and patient responses might leave room for subjective interpretation (e.g. MS disease course). Claims data come with their own limitations.^16^ The relevant health services and service providers are identified in our data, as universal health insurance exists in Germany.^7^ Services or providers that are not reimbursed or are reimbursed by other social welfare systems are not included in this analysis. We tried to focus only on MS-related health services. However, across sectors and health services, the diagnosis might be unavailable in claims data, not reliably coded (e.g. psychiatrist or psychotherapists might code MS diagnoses less often), or not exclusively related to health service utilisation due to MS (e.g. office-based physicians, inpatient care) (Table A1). Similarly, due to potentially incorrect coding in claims data, we may have missed eligible participants or mistakenly included non-eligible study participants. Despite non-responder analysis (Table A2), non-response bias remains possible.

We analysed PwMS ensured by a single insurance company (AOK Lower Saxony). Health insurance is mandatory in Germany,^7^ with 88.3% of the German population insured by a statutory health insurance company, such as AOK Lower Saxony.^35^ In 2017, regarding sex, the population insured by AOK Lower Saxony was comparable to the population living in Germany. The proportion of people aged ∼30 years or younger was higher, while the proportion of people between ∼50 and ∼76 years of age was lower. Older females (>80 years) were overrepresented in the population insured by AOK Lower Saxony. More specialised occupations were less common among those insured by AOK Lower Saxony than in the German population overall.^36^

MS-related health service utilisation has been analysed before in Germany^2,6,37^ and internationally,^4,38^ but services were evaluated individually^2,4,6,37,38^ or with a focus on definite study populations.^13,38^ Findings by Müller et al.^6^ regarding the utilisation of occupational therapy (∼9%) are comparable to our results, yet we identified a greater proportion of PwMS utilising physiotherapy (∼41% vs. ∼31%). About 60% of our study population received immunomodulatory treatment, which is consistent with other studies.^37,39^ For main health services that were analysed in both studies, Flachenecker et al.^39^ found similar proportions of health service utilisation, though focussing on shorter time periods. Only the proportion of PwMS consulting a GP for their MS needs is by far higher in our study (∼92% vs. ∼35%). Beckerman et al.^4^ adapted a previous version of Andersen's Behavioural Model.^10,11^ Using primary data, they identified neurologists as the physician most frequented by PwMS. The current analysis indicates that the GP is the most frequently visited outpatient physician, with outpatient neurologists being second. Yet, we suppose overestimation of GP utilisation in our data due to intensive coding of MS diagnosis in the context of primary care, while office-based GPs are the first line of contact for a large number of medical matters.

For the first time, we identified MS-related healthcare utilisation schemes and visualised a transition from *special services users* to *regular users* and ultimately to *assistive care users* as the disease progresses. Further analyses are required to confirm our results, to take into account possible contextual factors and to assess the appropriateness of care utilisation regarding care needs, as the present study solely describes actual care utilisation.

## Supplemental Material

sj-pdf-1-mso-10.1177_20552173241260151 - Supplemental material for Subgroup analyses and patterns of multiple sclerosis health service utilisation: A cluster analysis Supplemental material, sj-pdf-1-mso-10.1177_20552173241260151 for Subgroup analyses and patterns of multiple sclerosis health service utilisation: A cluster analysis by Lara Marleen Fricke, Kathrin Krüger, Corinna Trebst, Anna Levke Brütt, Elise-Marie Dilger, Kerstin Eichstädt, Peter Flachenecker, Anja Grau, Melissa Hemmerling, Dyon Hoekstra, Kristina Schaubert, Alexander Stahmann, Jona Theodor Stahmeyer, Annett Thiele, Uwe Klaus Zettl, Fedor Heidenreich and Christian Krauth in Multiple Sclerosis Journal – Experimental, Translational and Clinical

sj-docx-2-mso-10.1177_20552173241260151 - Supplemental material for Subgroup analyses and patterns of multiple sclerosis health service utilisation: A cluster analysis Supplemental material, sj-docx-2-mso-10.1177_20552173241260151 for Subgroup analyses and patterns of multiple sclerosis health service utilisation: A cluster analysis by Lara Marleen Fricke, Kathrin Krüger, Corinna Trebst, Anna Levke Brütt, Elise-Marie Dilger, Kerstin Eichstädt, Peter Flachenecker, Anja Grau, Melissa Hemmerling, Dyon Hoekstra, Kristina Schaubert, Alexander Stahmann, Jona Theodor Stahmeyer, Annett Thiele, Uwe Klaus Zettl, Fedor Heidenreich and Christian Krauth in Multiple Sclerosis Journal – Experimental, Translational and Clinical
