# Supplementary material for: Subgroup analyses and patterns of multiple sclerosis health service utilisation: A cluster analysis
Source: Mult Scler J Exp Transl Clin. 2024 Jun 20;10(2):20552173241260151. doi: 10.1177/20552173241260151 (PMC11191614; doi:10.1177/20552173241260151)
Supplement: sj-pdf-1-mso-10.1177_20552173241260151 - Supplemental material for Subgroup analyses and patterns of multiple sclerosis health service utilisation: A cluster analysis [file sj-pdf-1-mso-10.1177_20552173241260151.pdf]

Table A1. Overview of variables

Andersen's Behavioural Model[1]	Origin of variable or entire scale	Definition or modification of variable
Predisposing		
Age	Health insurance claims data	Ordinal scaled as suggested[2] or calculated as follows: 2021 – year of birth.
Sex	Health insurance claims data	Nominal scaled
Mother tongue	Online survey, item adapted[3,4]	Nominal scaled
Educational status	Online survey, item adapted[3,4]	Ordinal scaled as suggested, based on quintiles[5,6].<br>• low: value ≤ 3.0<br>• moderate: value > 3.0 to ≤ 4.8<br>• high: value > 4.8
Employment status	Online survey, item adapted[3,4]	Nominal scaled
Reduced earning capacity pension	Online survey, item adapted[7,8]	Nominal scaled
Children in household	Online survey, item adapted[3,4]	Ordinal scaled
Marital status	Online survey, item adapted[3,4]	Nominal scaled
Control preference	Online survey, Control Preference Scale (CPS), item adapted[9]	Ordinal scaled as suggested[10,11].
General Self-Efficacy	Online survey, General Self-Efficacy (GSE) scale[12,13]	Ordinal scaled according to the median which is 29, as suggested[14].
Enabling		
Household net income	Online survey, item adapted[3,4]	Assignment of random values within the selected groups and ordinal scaled according to the median of risk of poverty.[15,16]<br>Median net household income for German population in 2021.[17]<br>• risk of poverty: below 60% of monthly equivalised net income (2084.58€) = 1250.75€<br>• no risk of poverty: at least 60% of monthly equivalised net income (2084.58€) = 1250.75€
Region of residence	Health insurance claims data	Ordinal scaled, types of regions collapsed into 3 groups: "urban" (type 1 and 2), "suburban" (type 3 and 4) and "rural" (type 5).[18,19]
Main medical service provider for multiple sclerosis (self-identified)	Online survey, item self-developed	Nominal scaled.

Andersen's Behavioural Model[1]	Origin of variable or entire scale	Definition or modification of variable
		Question: Where does the main medical care for your multiple sclerosis (MS) currently take place? (single choice)<br>Answers: Outpatient general practitioner; outpatient neurologist; specialised MS center (e.g., outpatient department at a hospital); other; I do not receive medical care for my MS.
Living situation	Online survey, item adapted[20,21]	Nominal scaled
Informal care	Online survey, item adapted[22]	Nominal scaled, at least 1 day of informal care is defined as "support received".
Social support	Online survey, Brief Social Support (BS6) scale[23]	Ordinal scaled as suggested[23].
Self-help organisation	Online survey, item self-developed	Question: Are you currently a member of a self-help organisation or group for MS? (single choice)<br>Answers: Yes, rather active member; yes, rather passive member; no.<br>For analyses the options were further collapsed into "member" and "no member".
Need		
Multiple sclerosis related disability	Online survey, Patient Determined Disease Steps (PDDS) scale[24–27]	Ordinal scaled as suggested[28].<br>• mild: score 0 to 2<br>• moderate: score 3 to 5<br>• severe: score 6 to 8
Duration of multiple sclerosis	Online survey, item self-developed	Question: When did you receive your MS diagnosis? (single choice)<br>Answers: Select a year from 1940 to 2021 or chose "unknown".<br>For analyses the duration since diagnosis was calculated as follows: 2021 minus selected year and grouped as presented[29].
Time from symptoms onset to diagnosis	Online survey, item self-developed	Difference of question: When did you receive your MS diagnosis? (single choice) – question: In retrospect, when did symptoms first appear that you would attribute to MS? (single choice).<br>Answers: Select a year from 1940 to 2021 or chose "unknown".<br>For analyses the difference was grouped as inspired[30], page 49.
Course of multiple sclerosis	Online survey, item self-developed	Question: In general, different courses of MS can be distinguished. To which course would you most likely assign your MS, as of today? My MS course

Andersen's Behavioural Model[1]	Origin of variable or entire scale	Definition or modification of variable
		most closely resembles the following course: (single choice, schematic illustration of the courses provided)<br>Answers: The relapsing-remitting (SR) course; the secondary chronic progressive (SP) course without relapses; the secondary chronic progressive (SP) course with relapses; the primary chronic progressive (PP) course; I do not know.<br>For analyses the answers were further collapsed into nominal scaled options: "relapsing" (the relapsing-remitting (SR) course and the secondary chronic progressive (SP) course with relapses), "non-relapsing" (the secondary chronic progressive (SP) course without relapses and the primary chronic progressive (PP) course) and "unknown" (I do not know).
Comorbidity (CCI)	Health insurance claims data, Charlson Comorbidity index (CCI)[31]	Ordinal scaled as suggested[32].
Recent disease impairment	Online survey, item adapted[33]	Question: Please compare your state of health today with that of 24 months ago. Has the impairment due to MS become significantly more severe? Examples: Walking distance reduced? Everyday activities are no longer possible? (single choice)<br>Answers: Yes, significantly increased (e.g., significantly more problems); yes, somewhat increased (e.g., more problems); no, remained the same; slightly decreased (e.g., fewer problems); significantly decreased (e.g., significantly fewer problems).<br>For analyses the answers were further collapsed into ordinal scaled options: "more" (yes, significantly increased and yes, somewhat increased), "same" (no, remained the same), "less" (slightly and significantly decreased)
Relapses during past 24 months	Online survey, item self-developed	Question: How many relapses have occurred in the last 24 months?<br>Note: A relapse is only present when new symptoms or worsening of existing symptoms persist for at least 24 hours and other causes of worsening such as fever, infection, or high outside temperatures have been ruled out. New magnetic resonance imaging (MRI) changes alone are not a relapse. To speak of a second relapse, there must be an interval of at least 30 days from the onset of the last relapse. (single choice)<br>Answers: Select a number from 0 to 24 or chose "unknown".

Andersen's Behavioural Model[1]	Origin of variable or entire scale	Definition or modification of variable
		For analyses the answers were further collapsed into nominal scaled options: "none" (select 0) and "one or more" (select 1 or more)
Level of nursing care	Health insurance claims data	Nominal scaled as suggested[34]: "no or little impairment" (level 0 and 1) and "significant impairment" to independent living (level 2 to 5).
**Health Behaviours**		
Use of personal health services		
Outpatient care within previous 12 months (office-based physicians)	Health insurance claims data	• Timeframe October 1st, 2020, until September 30th, 2021<br>• Contact dates with a confirmed diagnosis International Classification of Diseases (ICD-10) of multiple sclerosis (G35.-)<br>• Number of contacts per study participant on which services were charged for<br>• Contacts vary in scope (e.g., laboratory (blood sampling by medical assistants following delegation) or instrumental diagnostics (electrocardiogram), comprehensive consultation with a doctor)
General practitioner		• Identification number of specialised physician group: 1, 2, 3
Neurologist		• Identification number of specialised physician group: 51, 53
Psychiatrist or psychotherapist		• Identification number of specialised physician group: 58, 60, 61, 68
Ophthalmologist		• Identification number of specialised physician group: 5
Dermatologist		• Identification number of specialised physician group: 21
Gynecologist		• Identification number of specialised physician group: 15, 16, 17
Urologist		• Identification number of specialised physician group: 67
Orthopedist		• Identification number of specialised physician group: 10, 12
Radiologist		• Identification number of specialised physician group: 54, 62, 64
Outpatient hospital based services		• Timeframe October 1st, 2020, until September 30th, 2021<br>• Count of days per study participant on which services were initialized<br>• Included are services according to § 117 SGB V: complex services conducted by outpatient clinics at the university hospitals "Hochschulambulanzen" and §116b SGB V: highly specialised outpatient services conducted by specialised hospital personnel "Hochspezialisierte Leistungen", a referral from an outpatient doctor is required for access

Andersen's Behavioural Model[1]	Origin of variable or entire scale	Definition or modification of variable
Inpatient care within the previous 12 months	Health insurance claims data	• Timeframe October 1st, 2020, until September 30th, 2021<br>• Multiple sclerosis primarydiagnosis (ICD-10) for inpatient clinical care<br>• Count of hospitalised days per study participant (day of admission and release counted as a full hospitalised day)
Inpatient care		
Rehabilitation within previous 12 months	Health insurance claims data	• Timeframe October 1st, 2020, until September 30th, 2021<br>• Count of days within timeframe of rehabilitation services per study participant (first and last day of timeframe inclusive)<br>• Full-time outpatient and inpatient rehabilitation services<br>• Rehabilitation services with a coded diagnosis (ICD-10) of multiple sclerosis (G35.-)
In- and outpatient rehabilitation		
Allied healthcare professionals within previous 12 months	Health insurance claims data	• Timeframe October 1st, 2020, until September 30th, 2021<br>• Allied healthcare professionals services with a coded diagnosis (ICD-10) of multiple sclerosis (G35.-)<br>• Only primary services provided prescribed for indication multiple sclerosis<br>• Count of primary service units per study participant on which services were charged
Physiotherapist		• Identification number for physiotherapists (German: "Heilmittelpositionsnummer" 2)<br>- Diagnosis group "ZN": service numbers 20501, 20507, 20710, 20711, 20712, 20902<br>- Diagnosis group "WS": service numbers 20106, 20107, 20501, 20507, 20902, 21201, 22001<br>- Diagnosis group "SO2" or "SO3": service numbers 20501, 20507<br>- Diagnosis group "PN": service numbers 20501, 20507<br>- Diagnosis group "LY": service numbers 20201, 20202, 20204, 20205<br>- Diagnosis group "EX": service numbers 20106, 20107, 20501, 20507, 20902, 21201<br>- Diagnosis group "CS": service numbers 20106, 20107, 20501, 20507, 20902

Andersen's Behavioural Model[1]	Origin of variable or entire scale	Definition or modification of variable
		- Diagnosis group "ATA": service numbers 20107, 20501, 20507<br>- Diagnosis group "AT2a": service numbers 20501, 20507
Occupational therapist		• Identification number for occupational therapist (German: "Heilmittelpositionsnummer" 5)<br>- Identification „EN1": service numbers 54102, 54103, 54104, 54105, 54210<br>- Diagnosis group "EN2": service numbers 54102, 54103, 54105, 54210<br>- Diagnosis group "EN3": service numbers 54102, 54103, 54210<br>- Diagnosis group "EN4": service numbers 54102, 54103, 54210<br>- Diagnosis group "PS2": service number 54105<br>- Diagnosis group "PS3": service number 54104, 54105<br>- Diagnosis group "PS4": service numbers 54104, 54105<br>- Diagnosis group "PS5": service numbers 54104, 54105<br>- Diagnosis group "SB1": service number 54102<br>- Diagnosis group "SB2": service numbers 54102, 54103, 54210<br>- Diagnosis group "SB3": service numbers 54102, 54103, 54210<br>- Diagnosis group "SB5": service number 54102<br>- Diagnosis group "SB7": service numbers 54102, 54103, 54210
Nursing care within previous 12 months	Health insurance claims data	• Timeframe October 1st, 2020, until September 30th, 2021<br>• Whether a study participant received nursing care services (either inpatient, outpatient or care allowance)
Nursing care services		
Medication within the previous 12 months	Health insurance claims data	• Timeframe October 1st, 2020, until September 30th, 2021<br>• Whether for a study participant was prescribed immunomodulatory therapy<br>• Definition of immunomodulatory therapy in outpatient prescriptions (Anatomical Therapeutic Chemical (ATC-) Code) independent of ICD-10 diagnosis<br>- L04AA31: Teriflunomide<br>- L03AB07: Interferon beta-1a<br>- L03AB08: Interferon beta-1b<br>- L03AX13: Glatiramer acetate

Andersen's Behavioural Model[1]	Origin of variable or entire scale	Definition or modification of variable
		- L04AX07: Dimethyl fumarate<br>- L01AA01: Cyclophosphamide<br>- L04AA27: Fingolimod<br>- L04AX01: Azathioprine<br>- L04AA52: Ofatumumab<br>- L04AA34: Alemtuzumab<br>- L01FA01: Rituximab<br>- L04AA40: Cladribine<br>- L04AA42: Siponimod<br>- L01DB07: Mitoxantrone<br>- L04AA36: Ocrelizumab<br>- L04AA50: Ponesimod<br>- L03AB13: Peginterferon beta-1a<br>- L04AA23: Natalizumab<br>- L04AX09: Diroximel fumarate<br>- L04AA38: Ozanimod<br>- L04AC01: Daclizumab<br>• Definition of immunomodulatory therapy in inpatient data (OPS code, German: "Operationen- und Prozedurenschluessel") coded with any ICD-10 diagnosis of multiple sclerosis<br>- 6-006.4: Ofatumumab, parenterally<br>- 6-001.h: Rituximab, intravenously<br>- 6-00a.4: Cladribine, orally<br>- 6-00a.e: Ocrelizumab, parenterally<br>- 6-003.f: Natalizumab, parenterally<br>- 6-009.9: Daclizumab, parenterally
Immunomodulatory therapy		
MRI scan within previous 12 months	Health insurance claims data	• Timeframe October 1st, 2020, until September 30th, 2021<br>• Whether a study participant received a MRI procedure<br>- Outpatient services: procedures with a confirmed diagnosis (ICD-10) of multiple sclerosis (G35.-) coded, MRI of the neurocranium or parts

Andersen's Behavioural Model[1]	Origin of variable or entire scale	Definition or modification of variable
		of the spine (EBM-catalogue, German: "Einheitlicher Bewertungsmaßstab": 34410, 34411)<br>- Outpatient hospital based services: MRI of the neurocranium or parts of the spine (EBM-catalogue, German: "Einheitlicher Bewertungsmaßstab": 34410, 34411)<br>- Inpatient services: Multiple sclerosis as main diagnosis (ICD-10) for inpatient clinical care or diagnosis (ICD-10) for pre-stationary services. MRI of the cranium and spine with and without contrast agents (OPS code, German: "Operationen- und Prozedurenschluessel": 380, 3800, 3801, 3802, 382, 3820, 3821, 3823)
MRI		
Mobility related assistive devices	Online survey, item self-developed	• Possession of<br>- Wheelchair (manual or electric) or walker<br>Question: Mobility-related assistive devices are relevant to the daily lives of many people with MS. Which of the following assistive devices do you own? (multiple choice)<br>Answers: Manual wheelchair; electric wheelchair; walker.
Possession of mobility related assistive devices		

Abbreviations: Brief Social Support scale (BS6); Charlson Comorbidity index (CCI); Control Preference Scale (CPS); German: "Einheitlicher Bewertungsmaßstab" (EBM-catalogue); General Self-Efficacy (GSE); International Classification of Diseases (ICD-10); magnetic resonance imaging (MRI); multiple sclerosis (MS); German: "Operationen- und Prozedurenschluessel" (OPS-code); Patient Determined Disease Steps (PDDS); the primary chronic progressive MS course (PP); the secondary chronic progressive MS course (SP); the relapsing-remitting MS course (SR).

Table A2. Non-responder analyses[35]

Variables within Andersen's Behavioural Model[1,36]		Sample: Respondents online survey		Sample: Entire sample		Pearson's Chi-squared test[37]	Cohen's W[#38]
		**n**	**%**	**n**	**%**	**p-value**	**effect size**
**Sample**		1,935	100.0	6,928	100.0		
**Individual characteristics**							
Predisposing							
Age						<0.001	0.129
	18-35 years	293	15.1	882	12.7		
	36-50 years	661	34.2	1,886	27.2		
	51-65 years	820	42.4	2,795	40.3		
	66 years and older	161	8.3	1,365	19.7		
Sex						0.2	0.014
	female	1,374	71.0	4,812	69.5		
	male	561	29.0	2,116	30.5		
Enabling							
Region of residence						0.9	0.004
	urban	393	20.3	1,407	20.3		
	suburban	655	33.9	2,375	34.3		
	rural	887	45.8	3,146	45.4		
Need							
Comorbidity (CCI)						<0.001	0.072
	none	970	50.1	3,034	43.8		
	1 to 2	640	33.1	2,335	33.7		
	3 to 4	243	12.6	1,019	14.7		
	5 or more	82	4.2	540	7.8		
Level of nursing care						<0.001	0.078
	no or little impairment	1,514	78.2	4,831	69.7		
	significant impairment	421	21.8	2,097	30.3		
**Utilisation of personal health services during a 12 months period (yes)**							

Outpatient care (office-based physicians)							
	General practitioner	1,789	92.5	6,090	87.9	<0.001	0.060
	Neurologist	1,652	85.4	4,809	69.4	<0.001	0.148
	Psychiatrist or psychotherapist	100	5.2	296	4.3	0.1	0.018
	Ophthalmologist	422	21.8	1,169	16.9	<0.001	0.053
	Dermatologist	113	5.8	296	4.3	0.004	0.031
	Gynaecologist	527	27.2	1,301	18.8	<0.001	0.086
	Urologist	268	13.9	845	12.2	0.06	0.021
	Orthopaedist	254	13.1	702	10.1	<0.001	0.040
	Radiologist	703	36.3	1,746	25.2	<0.001	0.103
	Outpatient hospital based services	175	9.0	482	7.0	0.002	0.033
Inpatient care							
	Inpatient care	165	8.5	454	6.6	0.003	0.032
Rehabilitation							
	In- and outpatient rehabilitation	84	4.3	212	3.1	0.007	0.029
Allied healthcare professionals							
	Physiotherapist	805	41.6	2,335	33.7	<0.001	0.068
	Occupational therapist	179	9.3	522	7.5	0.02	0.026
Care							
	Nursing care services	417	21.6	2,055	29.7	<0.001	0.075
Medication							
	Immunomodulatory therapy	1,173	60.6	3,068	44.3	<0.001	0.135
MRI scan							
	MRI	754	39.0	1,906	27.5	<0.001	0.103

[#]effect size: small 0.10 to <0.30, moderate: 0.30 to <0.50, large: ≥0.50.
Abbreviations: Charlson Comorbidity index (CCI); magnetic resonance imaging (MRI).

Table A3. Description of identified clusters of healthcare utilisation in four-cluster solution by complete linkage

Cluster	regular users (n=1,130)		assistive care users (n=443)		low users (n=195)		special services users (n=35)		Fisher's exact test[37]	Cramer's V[#38]
**Healthcare services**	**n**	**%**	**n**	**%**	**n**	**%**	**n**	**%**	**p-value**	**effect size**
**GP**									<0.001	0.143
no	85	7.5	19	4.3	35	17.9	1	2.9		
yes	1,045	92.5	424	95.7	160	82.1	34	97.1		
**NEURO**									<0.001	0.703
no	15	1.3	62	14.0	156	80.0	12	34.3		
yes	1,115	98.7	381	86.0	39	20.0	23	65.7		
**SPECIALISED CENTERS**									<0.001	0.357
no	1,044	92.4	405	91.4	188	96.4	7	20.0		
yes	86	7.6	38	8.6	7	3.6	28	80.0		
**OTHER OUTPATIENT**									<0.001	0.181
no	464	41.1	189	42.7	137	70.3	18	51.4		
yes	666	58.9	254	57.3	58	29.7	17	48.6		
**HOSPITAL**									<0.001	0.365
no	1,066	94.3	380	85.8	194	99.5	9	25.7		
yes	64	5.7	63	14.2	1	0.5	26	74.3		
**REHA**									0.003	0.080
no	1,076	95.2	423	95.5	194	99.5	31	88.6		
yes	54	4.8	20	4.5	1	0.5	4	11.4		
**THERAPISTS**									<0.001	0.453
no	719	63.6	89	20.1	182	93.3	17	48.6		
yes	411	36.4	354	79.9	13	6.7	18	51.4		
**CARE**									<0.001	0.834
no	1,112	98.4	86	19.4	192	98.5	34	97.1		
yes	18	1.6	357	80.6	3	1.5	1	2.9		
**DMT**									<0.001	0.493
no	261	23.1	238	53.7	188	96.4	7	20.0		
yes	869	76.9	205	46.3	7	3.6	28	80.0		

Cluster	regular users (n=1,130)		assistive care users (n=443)		low users (n=195)		special services users (n=35)		Fisher's exact test[37]	Cramer's V[#38]
**Healthcare services**	**n**	**%**	**n**	**%**	**n**	**%**	**n**	**%**	**p-value**	**effect size**
**MRI**									<0.001	0.328
no	592	52.4	300	67.7	191	97.9	3	8.6		
yes	538	47.6	143	32.3	4	2.1	32	91.4		
**AUX**									<0.001	0.837
no	1,060	93.8	27	6.1	180	92.3	30	85.7		
yes	70	6.2	416	93.9	15	7.7	5	14.3		

[#]effect size: small 0.06 to <0.17, moderate: 0.17 to <0.29, large: ≥0.29.

Abbreviations: office-based general practitioner (GP); office-based neurologist (NEURO); outpatient hospital based services (SPECIALISED CENTERS); office-based ophthalmologist, gynecologist, urologist orthopedist (OTHER OUTPATIENT); inpatient care (HOSPITAL); in- and outpatient rehabilitation (REHA); physiotherapist, occupational therapist (THERAPISTS); nursing care services (CARE); immunomodulatory therapy (DMT); magnetic resonance imaging (MRI); possession of mobility related assistive devices (AUX)

Table A4. Subgroup analyses of identified clusters of health service utilisation by complete linkage (non-imputed dataset)

Cluster	regular users (n=1,130)		assistive care users (n=443)		low users (n=195)		special services users (n=35)		Fisher's exact test[37]	Cramer's V[#38]
**Variables within Andersen model[4]**	**n**	**%**	**n**	**%**	**n**	**%**	**n**	**%**	**p-value**	**effect size**
Predisposing										
Age (years)		mean 46.6 SD 11.4		mean 56.6 SD 10.6		mean 51.0 SD 12.9		mean 42.7 SD 12.4	<0.001*	0.201
18-35	217	19.2	14	3.2	30	15.4	13	37.1		
36-50	454	40.2	103	23.3	54	27.7	12	34.3		
51-65	421	37.3	244	55.1	85	43.6	9	25.7		
66 and older	38	3.4	82	18.5	26	13.3	1	2.9		
Sex									0.1	0.054
female	822	72.7	304	68.6	135	69.2	21	60.0		
male	308	27.3	139	31.4	60	30.8	14	40.0		
Mother tongue									0.5	0.025
German	1,029	91.9	400	92.6	175	91.1	30	85.7		
other	91	8.1	32	7.4	17	8.9	5	14.3		
Educational status									<0.001*	0.113
low	238	21.4	174	40.7	63	32.6	6	18.8		
moderate	713	64.1	197	46.1	109	56.5	20	62.5		
high	100	9.0	26	6.1	11	5.7	4	12.5		
other	62	5.6	30	7.0	10	5.2	2	6.2		
Employment status									<0.001	0.342
employed	761	67.8	43	9.9	108	56.2	18	54.5		
not employed	361	32.2	390	90.1	84	43.8	15	45.5		
Reduced earning capacity pension									<0.001	0.271

Cluster	regular users (n=1,130)		assistive care users (n=443)		low users (n=195)		special services users (n=35)		Fisher's exact test[37]	Cramer's V[#38]
**Variables within Andersen model[4]**	**n**	**%**	**n**	**%**	**n**	**%**	**n**	**%**	**p-value**	**effect size**
without reduced earning capacity pension	826	73.8	134	31.2	147	77.0	26	78.8		
with reduced earning capacity pension	293	26.2	296	68.8	44	23.0	7	21.2		
Children in household									<0.001*	0.133
none	825	74.9	387	92.6	149	78.4	25	75.8		
one	140	12.7	14	3.3	18	9.5	6	18.2		
two or more	137	12.4	17	4.1	23	12.1	2	6.1		
Marital status									<0.001	0.087
serious relationship	918	81.8	315	72.1	133	69.3	26	74.3		
non-serious relationship	204	18.2	122	27.9	59	30.7	9	25.7		
Control preference (CPS)									<0.001*	0.102
active	453	40.3	145	34.1	82	55.8	17	50.0		
collaborative	565	50.3	209	49.2	54	36.7	14	41.2		
passive	105	9.3	71	16.7	11	7.5	3	8.8		
General Self-Efficacy (GSE)									<0.001	0.153
low self-efficacious	525	48.6	302	73.5	81	44.0	14	42.4		
high self-efficacious	555	51.4	109	26.5	103	56.0	19	57.6		
Enabling										
Household net income									<0.001	0.088
risk of poverty	390	37.9	210	53.0	82	46.6	13	43.3		
no risk of poverty	638	62.1	186	47.0	94	53.4	17	56.7		
Region of residence									0.1*	0.054
urban	230	20.4	89	20.1	43	22.1	4	11.4		
suburban	383	33.9	158	35.7	62	31.8	6	17.1		
rural	517	45.8	196	44.2	90	46.2	25	71.4		

Cluster	regular users (n=1,130)		assistive care users (n=443)		low users (n=195)		special services users (n=35)		Fisher's exact test[37]	Cramer's V[#38]
**Variables within Andersen model[4]**	**n**	**%**	**n**	**%**	**n**	**%**	**n**	**%**	**p-value**	**effect size**
Main medical service provider for multiple sclerosis (self-identified)									<0.001*	0.289
outpatient general practitioner	25	2.2	46	10.4	43	22.1	2	5.7		
outpatient neurologist	975	86.3	328	74.0	88	45.1	15	42.9		
specialised MS center	118	10.4	50	11.3	13	6.7	17	48.6		
other	6	0.5	2	0.5	3	1.5	0	0.0		
none	6	0.5	17	3.8	48	24.6	1	2.9		
Living situation									<0.001*	0.083
alone	166	14.7	95	21.7	52	26.8	7	20.6		
with family or spouse	936	82.9	319	73.0	138	71.1	24	70.6		
other	27	2.4	23	5.3	4	2.1	3	8.8		
Informal care									<0.001	0.379
no support received	807	72.6	51	11.8	154	80.2	23	65.7		
support received	304	27.4	382	88.2	38	19.8	12	34.3		
Social support (BS6)									<0.001*	0.085
low	160	14.3	36	8.4	43	22.6	5	14.7		
moderate	283	25.3	118	27.4	57	30.0	17	50.0		
high	445	39.8	183	42.5	57	30.0	8	23.5		
very high	229	20.5	94	21.8	33	17.4	4	11.8		
Self-help organisation									<0.001	0.097
member	153	13.6	109	24.8	21	10.8	5	14.3		
no member	974	86.4	331	75.2	174	89.2	30	85.7		
Need										

Cluster	regular users (n=1,130)		assistive care users (n=443)		low users (n=195)		special services users (n=35)		Fisher's exact test[37]	Cramer's V[#38]
**Variables within Andersen model[4]**	**n**	**%**	**n**	**%**	**n**	**%**	**n**	**%**	**p-value**	**effect size**
Multiple sclerosis related disability (PDDS)									<0.001*	0.396
mild	797	71.5	34	7.8	130	68.4	20	58.8		
moderate	300	26.9	174	39.9	55	28.9	13	38.2		
severe	18	1.6	228	52.3	5	2.6	1	2.9		
Duration of multiple sclerosis									<0.001*	0.172
less than 2 years	72	6.4	5	1.1	4	2.1	6	17.6		
2 to 15 years	697	62.0	155	35.3	105	54.1	19	55.9		
more than 15 years	337	30.0	273	62.2	81	41.8	9	26.5		
unknown	19	1.7	6	1.4	4	2.1	0	0.0		
Time from symptoms onset to diagnosis									<0.001*	0.079
up to 1 year	595	52.9	170	38.8	104	54.2	14	41.2		
2 to 6 years	261	23.2	124	28.3	44	22.9	10	29.4		
more than 6 years	172	15.3	103	23.5	28	14.6	8	23.5		
after diagnosis	20	1.8	10	2.3	3	1.6	1	2.9		
unknown	77	6.8	31	7.1	13	6.8	1	2.9		
Course of multiple sclerosis									<0.001*	0.158
relapsing	711	63.4	186	42.1	112	58.3	23	65.7		
non-relapsing	206	18.4	207	46.8	46	24.0	7	20.0		
unknown	205	18.3	49	11.1	34	17.7	5	14.3		
Comorbidity (CCI)									<0.001*	0.205
none	675	59.7	99	22.3	116	59.5	18	51.4		
1 to 2	333	29.5	194	43.8	53	27.2	16	45.7		
3 to 4	98	8.7	108	24.4	18	9.2	1	2.9		

Cluster	regular users (n=1,130)		assistive care users (n=443)		low users (n=195)		special services users (n=35)		Fisher's exact test[37]	Cramer's V[#38]
**Variables within Andersen model[4]**	**n**	**%**	**n**	**%**	**n**	**%**	**n**	**%**	**p-value**	**effect size**
5 or more	24	2.1	42	9.5	8	4.1	0	0.0		
Recent disease impairment									<0.001*	0.160
more	547	51.1	365	83.1	91	50.8	18	58.1		
same	463	43.3	65	14.8	79	44.1	11	35.5		
less	60	5.6	9	2.1	9	5.0	2	6.5		
Relapses during past 24 months									<0.001	0.073
none	599	53.7	197	45.4	103	52.8	8	23.5		
one or more	516	46.3	237	54.6	92	47.2	26	76.5		
Level of nursing care									<0.001	0.834
no or little impairment	1,111	98.3	84	19.0	191	97.9	34	97.1		
significant impairment	19	1.7	359	81.0	4	2.1	1	2.9		

Abbreviations: Brief Social Support scale (BS6); Charlson Comorbidity index (CCI); Control Preference Scale (CPS); General Self-Efficacy (GSE); number of patients (n); Patient Determined Disease Steps (PDDS), standard deviation (SD).

[#]effect size: small 0.06 to <0.17, moderate: 0.17 to <0.29, large: ≥0.29.

*Monte Carlo simulated p-value with 100,000 replicates

Highest value in comparison to variable characteristics in other clusters.

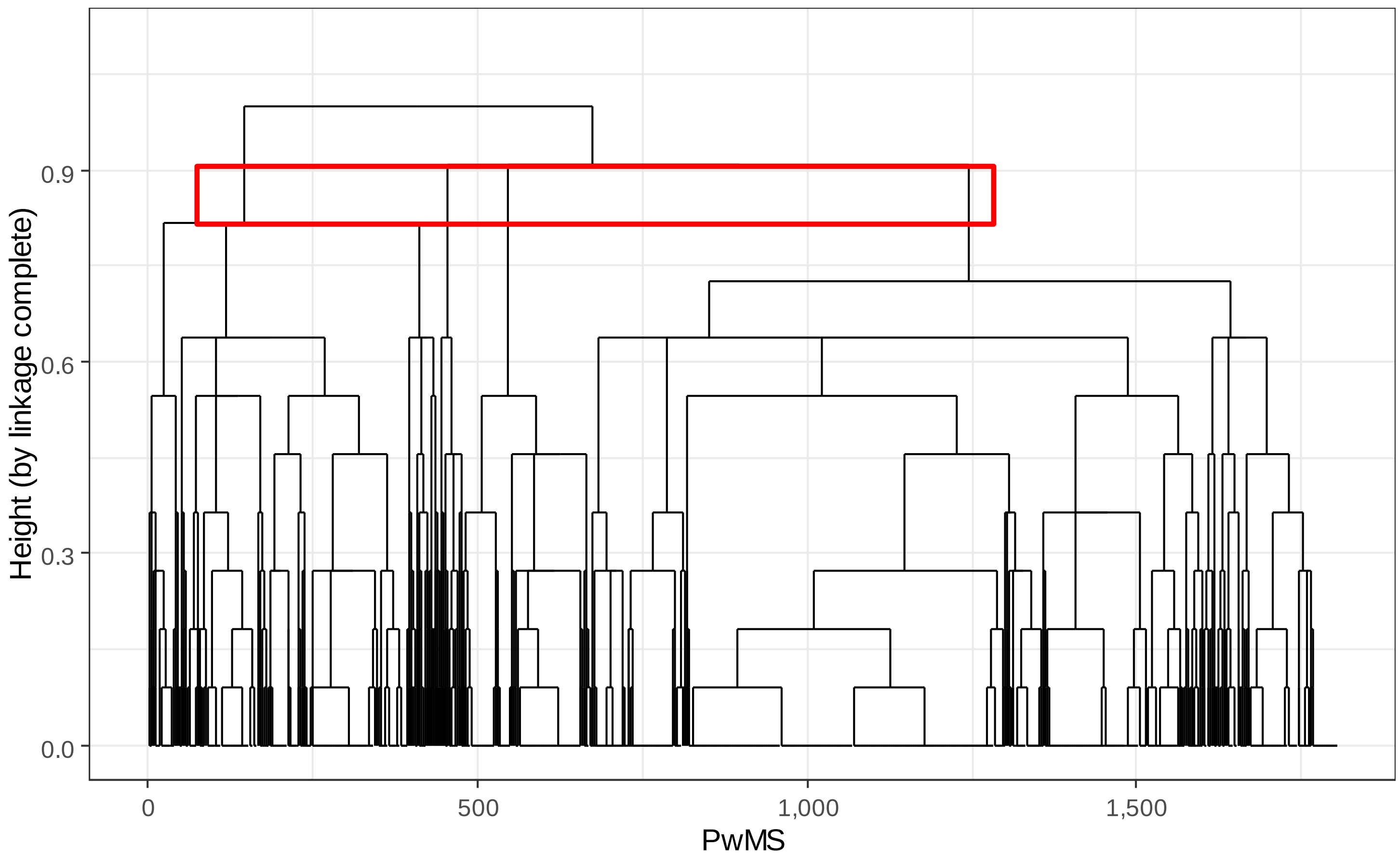

Figure A1. Dendrogram by complete linkage. *People with multiple sclerosis (PwMS).*

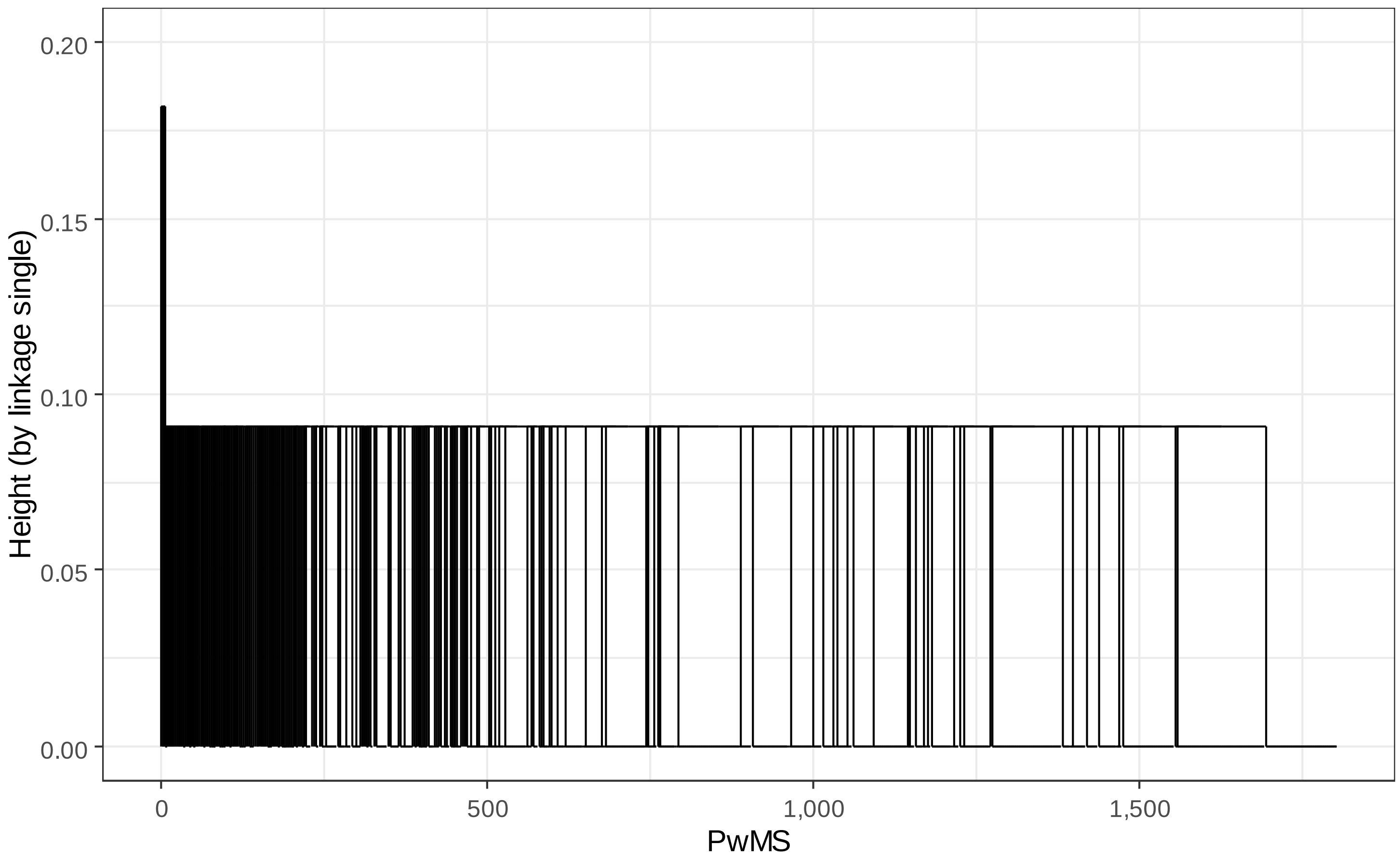

Figure A2. Dendrogram by single linkage. *People with multiple sclerosis (PwMS).*

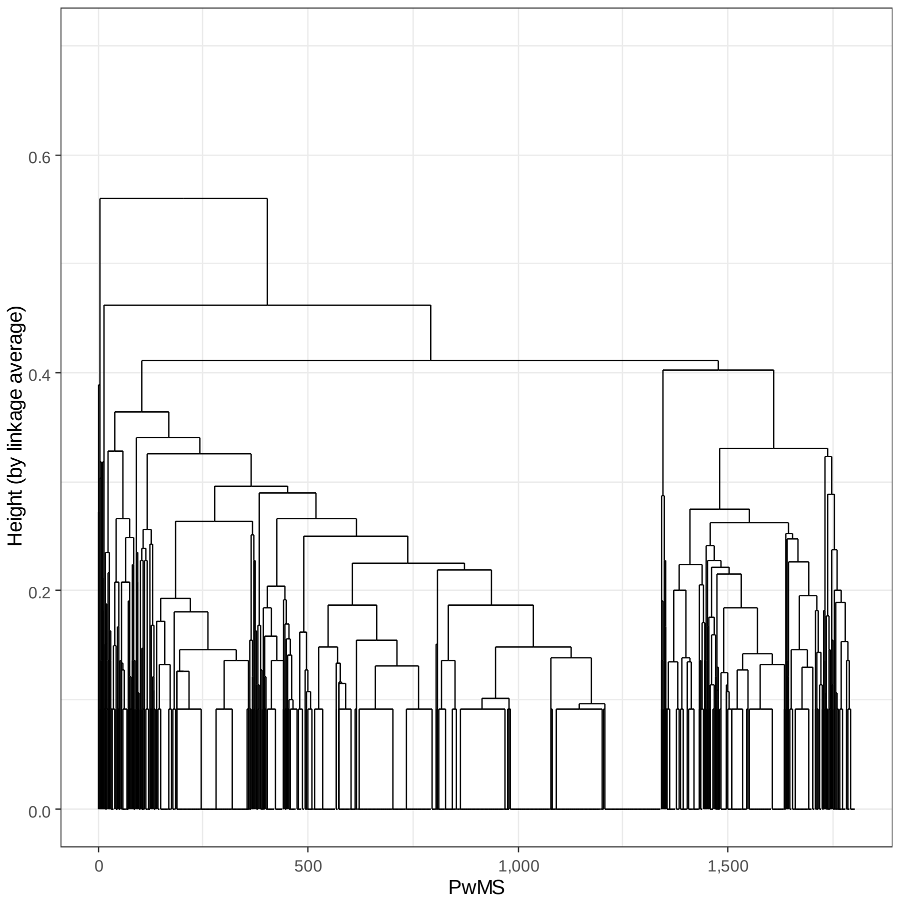

Figure A3. Dendrogram by average linkage. *People with multiple sclerosis (PwMS).*

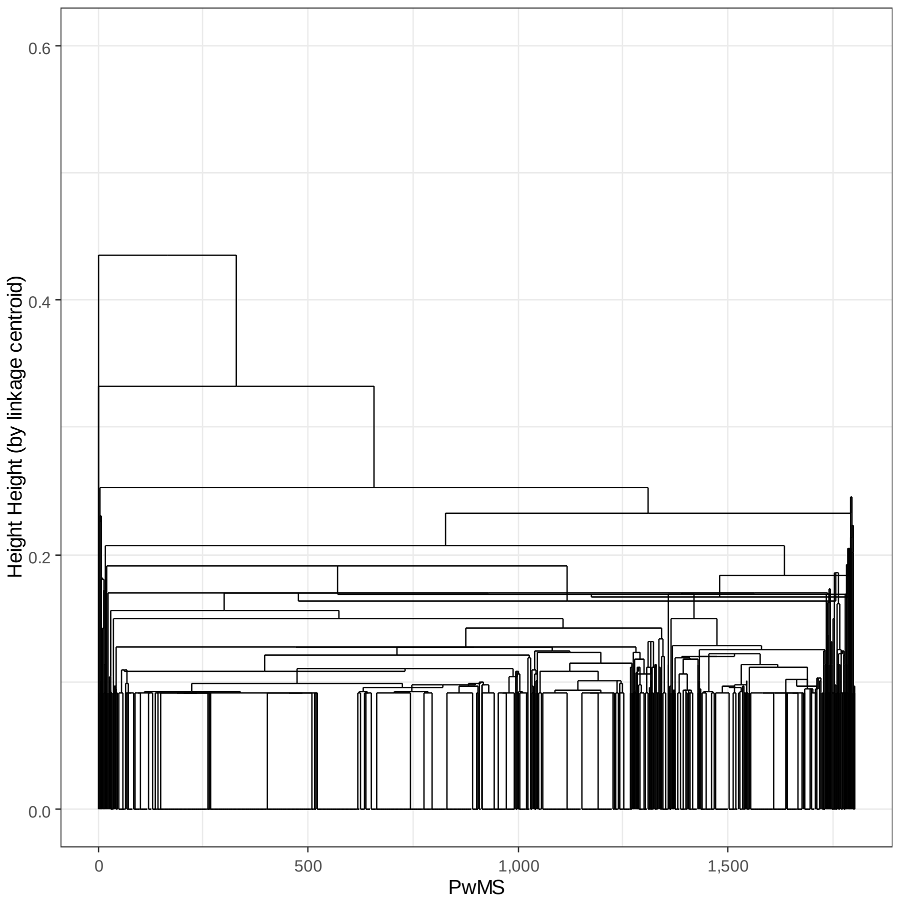

Figure A4. Dendrogram by centroid linkage. *People with multiple sclerosis (PwMS).*

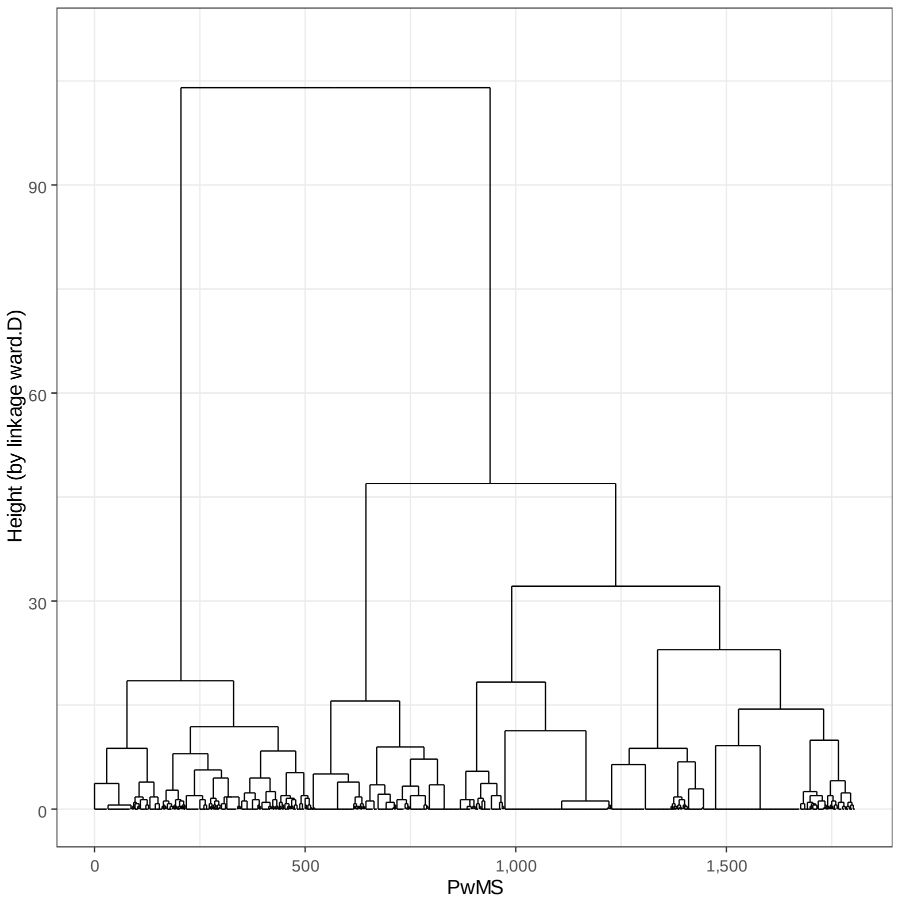

Figure A5. Dendrogram by ward linkage. *People with multiple sclerosis (PwMS).*

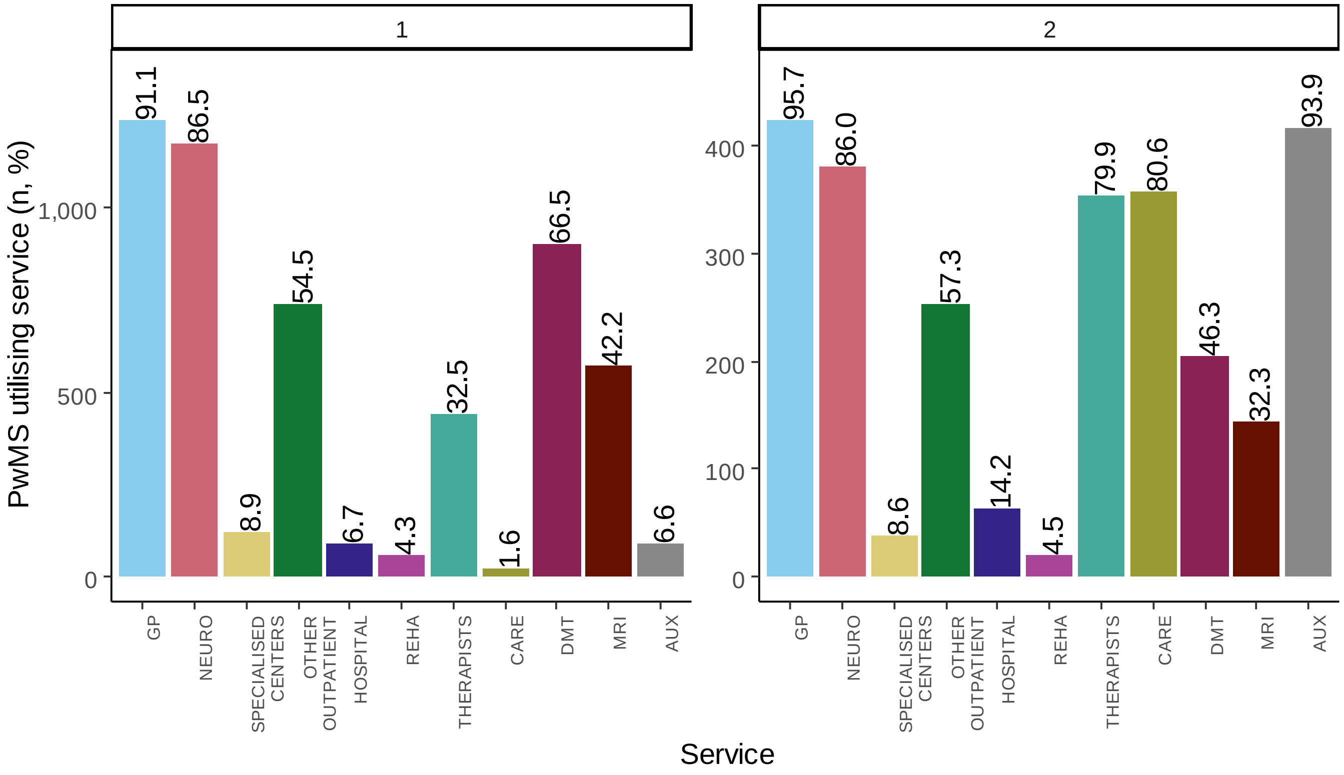

Figure A6. Identified clusters of healthcare utilisation of two-cluster solution by complete linkage. *People with multiple sclerosis (PwMS); office-based general practitioner (GP); office-based neurologist (NEURO); outpatient hospital based services (SPECIALISED CENTERS); office-based ophthalmologist, gynecologist, urologist orthopedist (OTHER OUTPATIENT); inpatient care (HOSPITAL); in- and outpatient rehabilitation (REHA); physiotherapist, occupational therapist (THERAPISTS); nursing care services (CARE); immunomodulatory therapy (DMT); magnetic resonance imaging (MRI); possession of mobility related assistive devices (AUX).*

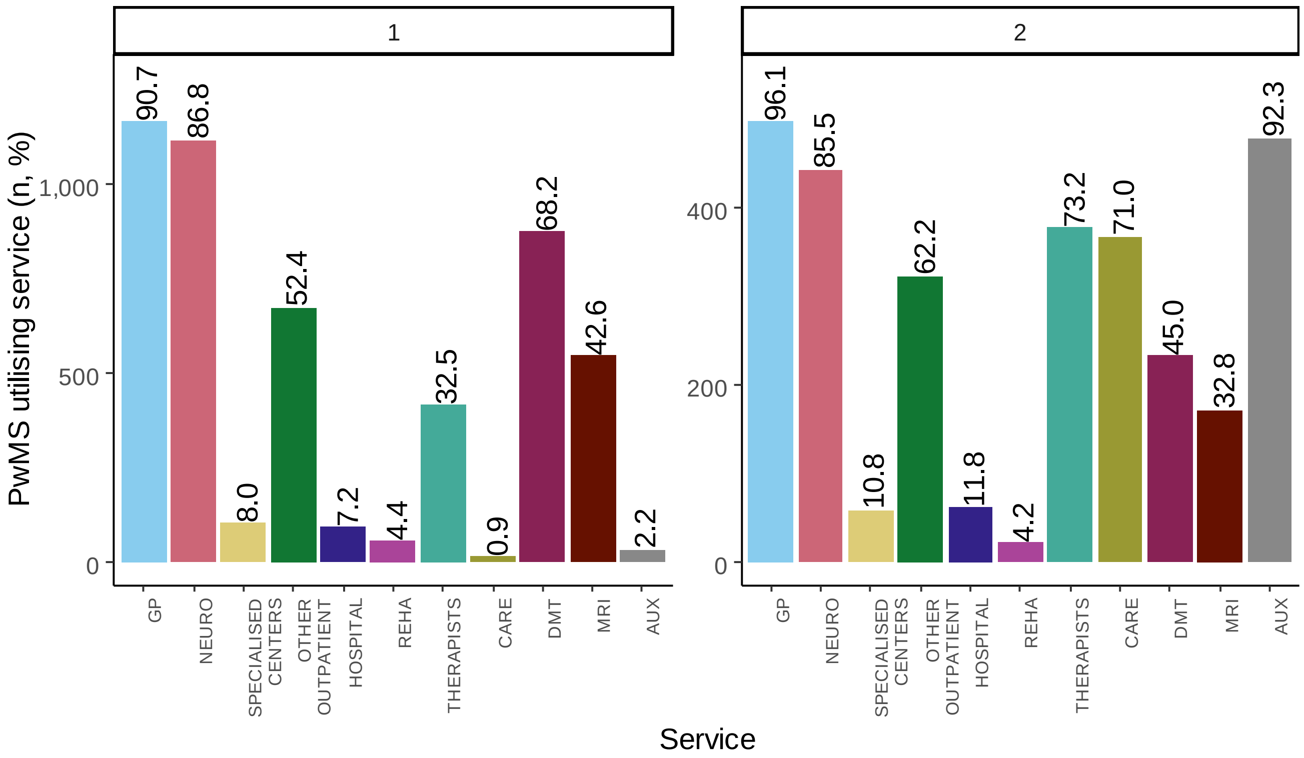

Figure A7. Identified clusters of healthcare utilisation of two-cluster solution by Ward.D linkage. *People with multiple sclerosis (PwMS); office-based general practitioner (GP); office-based neurologist (NEURO); outpatient hospital based services (SPECIALISED CENTERS); office-based ophthalmologist, gynecologist, urologist orthopedist (OTHER OUTPATIENT); inpatient care (HOSPITAL); in- and outpatient rehabilitation (REHA); physiotherapist, occupational therapist (THERAPISTS); nursing care services (CARE); immunomodulatory therapy (DMT); magnetic resonance imaging (MRI); possession of mobility related assistive devices (AUX).*

References

1. Andersen RM, Davidson PL and Baumeister SE. Improving access to care. In: Kominski GF (ed.) *Changing the U.S. Health Care System: Key Issues in Health Services Policy and Management*. 4 ed. Hoboken: Wiley, 2013, pp. 33–69.
2. Beckerman H, van Zee IE, Groot V de, et al. Utilization of health care by patients with multiple sclerosis is based on professional and patient-defined health needs. *Mult Scler* 2008; 14: 1269–1279.
3. Gößwald A, Lange M, Dölle R, et al. Die erste Welle der Studie zur Gesundheit Erwachsener in Deutschland (DEGS1): Gewinnung von Studienteilnehmenden, Durchführung der Feldarbeit und Qualitätsmanagement. *Bundesgesundheitsblatt Gesundheitsforschung Gesundheitsschutz* 2013; 56: 611–619.
4. Kamtsiuris P, Lange M, Hoffmann R, et al. Die erste Welle der Studie zur Gesundheit Erwachsener in Deutschland (DEGS1): Stichprobendesign, Response, Gewichtung und Repräsentativität. *Bundesgesundheitsblatt Gesundheitsforschung Gesundheitsschutz* 2013; 56: 620–630.
5. Laussmann D, Haftenberger M, Lampert T, et al. Soziale Ungleichheit von Lärmbelästigung und Straßenverkehrsbelastung: Ergebnisse der Studie zur Gesundheit Erwachsener in Deutschland (DEGS1). *Bundesgesundheitsblatt Gesundheitsforschung Gesundheitsschutz* 2013; 56: 822–831.
6. Lampert T, Kroll LE, Müters S, et al. Messung des sozioökonomischen Status in der Studie "Gesundheit in Deutschland aktuell" (GEDA). *Bundesgesundheitsblatt Gesundheitsforschung Gesundheitsschutz* 2013; 56: 131–143.
7. Flachenecker P, Kobelt G, Berg J, et al. New insights into the burden and costs of multiple sclerosis in Europe: Results for Germany. *Mult Scler* 2017; 23: 78–90.
8. Kobelt G, Thompson A, Berg J, et al. New insights into the burden and costs of multiple sclerosis in Europe. *Mult Scler* 2017; 23: 1123–1136, https://onlinelibrary.wiley.com/doi/pdf/10.1046/j.1365-2648.2003.02579.x (2017, accessed 17 May 2021).
9. Solari A, Giordano A, Kasper J, et al. Role preferences of people with multiple sclerosis: Image-revised, computerized self-administered version of the Control Preference Scale. *PLoS One* 2013; 8: e66127.
10. Becker C, Gross S, Gamp M, et al. Patients' Preference for Participation in Medical Decision-Making: Secondary Analysis of the BEDSIDE-OUTSIDE Trial. *J Gen Intern Med* 2022.
11. Cofield SS, Thomas N, Tyry T, et al. Shared Decision Making and Autonomy Among US Participants with Multiple Sclerosis in the NARCOMS Registry. *Int J MS Care* 2017; 19: 303–312.
12. Schwarzer R and Jerusalem M (eds). *Skalen zur Erfassung von Lehrer- und Schülermerkmalen: Dokumentation der psychometrischen Verfahren im Rahmen der Wissenschaftlichen Begleitung des Modellversuchs Selbstwirksame Schulen*. Berlin: Freie Universität Berlin, 1999.
13. Schwarzer R and Jerusalem M. Generalized Self-Efficacy scale. In: Weinman J, Wright SC and Johnston M (eds) *Measures in Health Psychology: A User's Portfolio. Causal and control beliefs*. Windsor, England: NFER - Nelson, 1995, pp. 35–37.
14. Schwarzer R. Everything you wanted to know about the General Self-Efficacy Scale but were afraid to ask: Documentation of the General Self-Efficacy Scale, http://userpage.fu-berlin.de/~health/faq_gse.pdf (2014).

15. Lampert T, Kroll L, Müters S, et al. Messung des sozioökonomischen Status in der Studie zur Gesundheit Erwachsener in Deutschland (DEGS1). *Bundesgesundheitsblatt Gesundheitsforschung Gesundheitsschutz* 2013; 56: 631–636.
16. Bundesministerium für Arbeit und Soziales. Lebenslagen in Deutschland: Der 3. Armuts- und Reichtumsbericht der Bundesregierung, Berlin, 2008.
17. Statistisches Bundesamt. Lebensbedingungen und Armutsgefährdung: Einkommensverteilung (Nettoäquivalenzeinkommen), https://www.destatis.de/DE/Themen/Gesellschaft-Umwelt/Einkommen-Konsum-Lebensbedingungen/Lebensbedingungen-Armutsgefaehrdung/Tabellen/einkommensverteilung-mz-silc.html (2022).
18. Bundesministerium für Gesundheit. *Bekanntmachung eines Beschlusses des Gemeinsamen Bundesausschusses über eine Änderung der Bedarfsplanungs-Richtlinie: Anpassung der Verhältniszahl für Kinder- und Jugendpsychiaterinnen und -psychiater*, 2022.
19. Kassenärztliche Bundesvereinigung. Typisierung der Bedarfsplanungsregionen, https://gesundheitsdaten.kbv.de/cms/html/17013.php (2023).
20. Ness N-H, Haase R, Kern R, et al. The Multiple Sclerosis Health Resource Utilization Survey (MS-HRS): Development and validation study. *J Med Internet Res* 2020; 22: e17921.
21. Ness N-H, Schriefer D, Haase R, et al. Der Multiple Sclerosis Health Resource Utilization Survey. *Fortschr Neurol Psychiatr* 2021.
22. Seidl H, Bowles D, Bock J-O, et al. FIMA--Fragebogen zur Erhebung von Gesundheitsleistungen im Alter: Entwicklung und Pilotstudie. *Gesundheitswesen* 2015; 77: 46–52.
23. Beutel ME, Brähler E, Wiltink J, et al. Emotional and tangible social support in a German population-based sample: Development and validation of the Brief Social Support Scale (BS6). *PLoS One* 2017; 12: e0186516.
24. Hohol MJ, Orav EJ and Weiner HL. Disease steps in multiple sclerosis: A simple approach to evaluate disease progression. *Neurology* 1995; 45: 251–255.
25. Hohol MJ, Orav EJ and Weiner HL. Disease steps in multiple sclerosis: A longitudinal study comparing disease steps and EDSS to evaluate disease progression. *Mult Scler* 1999; 5: 349–354.
26. Marrie RA and Goldman M. Validity of performance scales for disability assessment in multiple sclerosis. *Mult Scler* 2007; 13: 1176–1182.
27. Pöttgen J, Moss-Morris R, Wendebourg J-M, et al. Randomised controlled trial of a self-guided online fatigue intervention in multiple sclerosis. *J Neurol Neurosurg Psychiatry* 2018; 89: 970–976.
28. Nazareth TA, Rava AR, Polyakov JL, et al. Relapse prevalence, symptoms, and health care engagement: patient insights from the Multiple Sclerosis in America 2017 survey. *Mult Scler Relat Disord* 2018; 26: 219–234.
29. Stuke K, Flachenecker P, Zettl U, et al. MS-Register in Deutschland 2008: Symptomatik der MS. *Akt Neurol* 2008; 35.
30. Kip M, Schönfelder T and Bleß H-H. *Weißbuch Multiple Sklerose*. Berlin, Heidelberg: Springer Berlin Heidelberg, 2016.
31. Charlson ME, Pompei P, Ales KL, et al. A new method of classifying prognostic comorbidity in longitudinal studies: development and validation. *J Chronic Dis* 1987; 40: 373–383.

32. Chou IJ, Kuo CF, Tanasescu R, et al. Comorbidity in multiple sclerosis: its temporal relationships with disease onset and dose effect on mortality. *Eur J Neurol* 2020; 27: 105–112.
33. Heesen C, Köpke S, Kasper J, et al. Immuntherapien der Multiplen Sklerose 2008, Hamburg, 2008.
34. Gehr TJ, Freiberger E, Sieber CC, et al. A typology of caregiving spouses of geriatric patients without dementia: caring, worried, desperate. *BMC Geriatr* 2021; 21: 483.
35. Fricke LM, Krüger K, Schaubert K, et al. Non-Responder-Analyse der Eigenschaften der Teilnehmenden an einer Online-Befragung unter Multiple Sklerose Erkrankten der AOK Niedersachsen. In: *Gemeinsame Jahrestagung der Deutschen Gesellschaft für Sozialmedizin und Prävention e.V. (DGSMP), des Deutschen Netzwerks Gesundheitskompetenz e. V. (DNGK) und des Nationalen Aktionsplans Gesundheitskompetenz (NAP): Gesundheitskompetenz in Krisenzeiten*. (ed DGSMP, DNGK and NAP), Hannover, Germany, 30.08 – 01.09.2023, p. 814. Stuttgart, Germany: Georg Thieme Verlag.
36. Fricke LM, Krüger K, Schaubert K, et al. Non-Responder-Analyse auf Basis der in Routinedaten definierten Leistungsbereiche der Teilnehmenden an einer Online-Befragung zur Versorgungssituation von Multipler Sklerose in Niedersachsen. In: *22. Deutscher Kongress für Versorgungsforschung*. (ed Deutsches Netzwerk Versorgungsforschung e. V.), Berlin, Germany, 04.10. - 06.10.2023, GM23dkvf285: German Medical Science (GMS).
37. R Core Team. *R: A language and environment for statistical computing*. Vienna, Austria: R Foundation for Statistical Computing, 2023.
38. Mangiafico S. *rcompanion: Functions to Support Extension Education Program Evaluation*, 2023.
