# Supplementary material for: Subgroup analyses and patterns of multiple sclerosis health service utilisation: A cluster analysis
Source: Mult Scler J Exp Transl Clin. 2024 Jun 20;10(2):20552173241260151. doi: 10.1177/20552173241260151 (PMC11191614; doi:10.1177/20552173241260151)
Supplement: sj-docx-2-mso-10.1177_20552173241260151 - Supplemental material for Subgroup analyses and patterns of multiple sclerosis health service utilisation: A cluster analysis [file sj-docx-2-mso-10.1177_20552173241260151.docx]

# STROBE Statement—Checklist of items that should be included in reports of cross-sectional studies^1^

|  | Item No | Recommendation | Page No |
| --- | --- | --- | --- |
| **Title and abstract** | 1 | (*a*) Indicate the study’s design with a commonly used term in the title or the abstract | Title page; main document: abstract |
|  |  | (*b*) Provide in the abstract an informative and balanced summary of what was done and what was found | Main document: abstract |
| Introduction | | | |
| Background/rationale | 2 | Explain the scientific background and rationale for the investigation being reported | Main document: introduction |
| Objectives | 3 | State specific objectives, including any prespecified hypotheses | Main document: introduction |
| Methods | | | |
| Study design | 4 | Present key elements of study design early in the paper | Main document: materials and methods |
| Setting | 5 | Describe the setting, locations, and relevant dates, including periods of recruitment, exposure, follow-up, and data collection | Main document: materials and methods – study population, data sources |
| Participants | 6 | (*a*) Give the eligibility criteria, and the sources and methods of selection of participants | Main document: materials and methods – study population, |
| Variables | 7 | Clearly define all outcomes, exposures, predictors, potential confounders, and effect modifiers. Give diagnostic criteria, if applicable | Main document: materials and methods – variables; figure 1; table A1 |
| Data sources/ measurement | 8* | For each variable of interest, give sources of data and details of methods of assessment (measurement). Describe comparability of assessment methods if there is more than one group | Main document: materials and methods – data sources, variables; figure 1; table A1 |
| Bias | 9 | Describe any efforts to address potential sources of bias | Main document: materials and methods – study population, data sources, variables, data analyses; table A2 |
| Study size | 10 | Explain how the study size was arrived at | Main document: materials and methods – study population, data sources, variables (online survey) |
| Quantitative variables | 11 | Explain how quantitative variables were handled in the analyses. If applicable, describe which groupings were chosen and why | Table A1 |
| Statistical methods | 12 | (*a*) Describe all statistical methods, including those used to control for confounding | Main document: materials and methods –data analyses |
|  |  | (*b*) Describe any methods used to examine subgroups and interactions | Main document: materials and methods –data analyses |
|  |  | (*c*) Explain how missing data were addressed | Main document: materials and methods –data analyses |
|  |  | (*d*) If applicable, describe analytical methods taking account of sampling strategy | Not applicable |
|  |  | (*e*) Describe any sensitivity analyses | Main document: materials and methods –data analyses |
| Results | | | |
| Participants | 13* | (a) Report numbers of individuals at each stage of study—eg numbers potentially eligible, examined for eligibility, confirmed eligible, included in the study, completing follow-up, and analysed | Main document: results; figure 2 |
|  |  | (b) Give reasons for non-participation at each stage | Main document: results; figure 2 |
|  |  | (c) Consider use of a flow diagram | Figure 2 |
| Descriptive data | 14* | (a) Give characteristics of study participants (eg demographic, clinical, social) and information on exposures and potential confounders | Main document: results; table 1 |
|  |  | (b) Indicate number of participants with missing data for each variable of interest | Main document: results; table 1 |
| Outcome data | 15* | Report numbers of outcome events or summary measures | Main document: results; table 2 |
| Main results | 16 | (*a*) Give unadjusted estimates and, if applicable, confounder-adjusted estimates and their precision (eg, 95% confidence interval). Make clear which confounders were adjusted for and why they were included | Not applicable |
|  |  | (*b*) Report category boundaries when continuous variables were categorized | Table A1 |
|  |  | (*c*) If relevant, consider translating estimates of relative risk into absolute risk for a meaningful time period | Not applicable |
| Other analyses | 17 | Report other analyses done—eg analyses of subgroups and interactions, and sensitivity analyses | Main document: results ; table 3, figure 3; tables A3-4 |
| Discussion | | | |
| Key results | 18 | Summarise key results with reference to study objectives | Main document: discussion |
| Limitations | 19 | Discuss limitations of the study, taking into account sources of potential bias or imprecision. Discuss both direction and magnitude of any potential bias | Main document: discussion |
| Interpretation | 20 | Give a cautious overall interpretation of results considering objectives, limitations, multiplicity of analyses, results from similar studies, and other relevant evidence | Main document: discussion |
| Generalisability | 21 | Discuss the generalisability (external validity) of the study results | Main document: discussion |
| Other information | | | |
| Funding | 22 | Give the source of funding and the role of the funders for the present study and, if applicable, for the original study on which the present article is based | Main document: funding |

*Give information separately for exposed and unexposed groups.

References

1. Elm E von, Altman DG, Egger M, et al. The Strengthening the Reporting of Observational Studies in Epidemiology (STROBE) statement: guidelines for reporting observational studies. *Lancet* 2007; 370: 1453–1457.
